# Changes in the Proteome of Langat-Infected *Ixodes scapularis* ISE6 Cells: Metabolic Pathways Associated with Flavivirus Infection

**DOI:** 10.1371/journal.pntd.0004180

**Published:** 2016-02-09

**Authors:** Jeffrey M. Grabowski, Rushika Perera, Ali M. Roumani, Victoria E. Hedrick, Halina D. Inerowicz, Catherine A. Hill, Richard J. Kuhn

**Affiliations:** 1 Department of Entomology, College of Agriculture, Purdue University, West Lafayette, Indiana, United States of America; 2 Markey Center for Structural Biology, Department of Biological Sciences, College of Science, Purdue University, West Lafayette, Indiana, United States of America; 3 Bindley Bioscience Center, Purdue University, West Lafayette, Indiana, United States of America; George Mason University, UNITED STATES

## Abstract

**Background:**

Ticks (Family Ixodidae) transmit a variety of disease causing agents to humans and animals. The tick-borne flaviviruses (TBFs; family Flaviviridae) are a complex of viruses, many of which cause encephalitis and hemorrhagic fever, and represent global threats to human health and biosecurity. Pathogenesis has been well studied in human and animal disease models. Equivalent analyses of tick-flavivirus interactions are limited and represent an area of study that could reveal novel approaches for TBF control.

**Methodology/Principal Findings:**

High resolution LC-MS/MS was used to analyze the proteome of *Ixodes scapularis* (Lyme disease tick) embryonic ISE6 cells following infection with Langat virus (LGTV) and identify proteins associated with viral infection and replication. Maximal LGTV infection of cells and determination of peak release of infectious virus, was observed at 36 hours post infection (hpi). Proteins were extracted from ISE6 cells treated with LGTV and non-infectious (UV inactivated) LGTV at 36 hpi and analyzed by mass spectrometry. The Omics Discovery Pipeline (ODP) identified thousands of MS peaks. Protein homology searches against the *I*. *scapularis* IscaW1 genome assembly identified a total of 486 proteins that were subsequently assigned to putative functional pathways using searches against the Kyoto Encyclopedia of Genes and Genomes (KEGG) database. 266 proteins were differentially expressed following LGTV infection relative to non-infected (mock) cells. Of these, 68 proteins exhibited increased expression and 198 proteins had decreased expression. The majority of the former were classified in the KEGG pathways: “translation”, “amino acid metabolism”, and “protein folding/sorting/degradation”. Finally, Trichostatin A and Oligomycin A increased and decreased LGTV replication *in vitro* in ISE6 cells, respectively.

**Conclusions/Significance:**

Proteomic analyses revealed ISE6 proteins that were differentially expressed at the peak of LGTV replication. Proteins with increased expression following infection were associated with cellular metabolic pathways and glutaminolysis. *In vitro* assays using small molecules implicate malate dehydrogenase (MDH2), the citrate cycle, cellular acetylation, and electron transport chain processes in viral replication. Proteins were identified that may be required for TBF infection of ISE6 cells. These proteins are candidates for functional studies and targets for the development of transmission-blocking vaccines and drugs.

## Author Summary

High-throughput proteomics offers an approach to evaluate changes in cell protein levels following arboviral infection. Research to understand the molecular basis of human-flavivirus interactions has advanced significantly over the past decade, but comparatively little is known regarding interactions between ticks and tick-borne flaviviruses (TBFs). Here, we employed a proteomics approach using an *I*. *scapularis* ISE6 cell line infected with the TBF Langat virus (LGTV) to identify proteins and biochemical pathways affected by viral infection. An LC-MS/MS approach was used to identify proteins that were subsequently assigned to putative cellular pathways based on orthology to proteins in the KEGG database. Biochemical pathways common among arthropods in response to infection with flavivirus and possibly unique to tick-flavivirus interactions, were identified. *In vitro* cellular assays using small molecules suggest the involvement of the ISE6 proteins, malate dehydrogenase (MDH2), and mitochondria in viral replication. These analyses provide a basis for further studies to identify tick proteins associated with viral replication that could be targeted to disrupt TBF transmission.

## Introduction

Tick-borne flaviviruses (TBFs; family Flaviviridae) are a complex of positive, single-stranded RNA viruses, many of which cause hemorrhagic fever and encephalitis in humans and are associated with high morbidity and mortality [1, 2]. Humans are incidental hosts for TBFs that are transmitted by an infected tick (subphylum Chelicerata, subclass Acari; superfamily Ixodida) during blood feeding. Tick-borne encephalitis virus (TBEV) is the most prevalent TBF worldwide and is responsible for over 10,000 confirmed cases of encephalitis globally per annum [3, 4]. Several TBFs associated with hemorrhagic disease are identified on the Centers for Disease Control and Prevention (CDC) “Select Biological Agents and Toxins” list (http://www.selectagents.gov/) due to their high virulence (biosafety level 3 and 4), anticipated ability to establish zoonotic transmission cycles, and their potential use in bioterrorism. Of these, Kyasanur Forest Disease virus (KFDV) is responsible for an estimated 400–500 human cases per year in India [5–7] while Omsk hemorrhagic fever virus (OHFV) is estimated to cause an average of 24 human cases per year (1946–2000) [8]. In the U.S., The increasing incidence of human cases of Powassan virus (POWV) and the corresponding genotype virus, Deer Tick virus (DTV) [9, 10] in the northeast and upper mid-west of the U.S., has refocused attention on TBFs in North America.

Langat virus (LGTV) was discovered in Southeast Asia in the 1950s [11]. LGTV exhibits low levels of virulence to humans, is classified as biosafety level 2 (BSL2) and employed routinely as a model for more virulent TBFs such as TBEV, KFDV, OHFV, and POWV/DTV. Other than for TBEV [5, 12], there are no vaccines or therapeutics available to prevent or treat infection with these virulent TBFs. Globally, there is an urgent need to identify novel prophylactics and therapeutics against TBFs.

The NIH-funded *Ixodes scapularis* (Lyme disease tick) Genome Project represents the first genome assembly for a tick and an important resource to understand the molecular processes in ticks [13]. The IscaW1.2 annotation comprises 20,450 gene models predicted via a combination of *ab initio* methods and manual curation. These models are a source of new targets [14] for the identification of novel chemistries [15] and vaccines [16–18] for control of ticks and tick-borne diseases.

Research has shown that proteins and metabolites produced by human [19, 20] and mosquito [21–24] cells (i.e., “host-cell factors”) may facilitate or play essential roles in flaviviral infection [25–28]. The mechanisms by which these molecules contribute to the pathogenesis of the *Flaviviridae*, are not well understood. Proteomics has been used to investigate interactions between ticks and bacterial pathogens [29–31]. Studies have also investigated global changes in the transcriptome of *I*. *scapularis* and in tick cells following LGTV infection [32], although there is little known about how these responses correlate to changes at the protein level. Tick proteins that facilitate viral infection and replication in the arthropod vector are logical targets for interventions aimed at disrupting transmission of TBF. Here we developed an *in vitro* assay using the *I*. *scapularis* ISE6 embryonic cell line [33–35] and LGTV (TP21 wildtype strain). We performed high-resolution LC-MS/MS analyses to evaluate global changes in the proteome of tick cells following flavivirus infection and identified proteins that displayed increased and decreased expression. We describe the cellular response to infection and employ small molecule functional assays to evaluate the involvement of several tick proteins in the infection and replication of LGTV in ISE6 cells.

## Methods

### Cell and virus culture

*Ixodes scapularis* embryonic ISE6 cells (provided by T. Kurtti, University of Minnesota, Minneapolis, MN) were cultured at 34°C in L15B-300 medium in the absence of CO_2_ [36, 37]. Baby hamster kidney 15 (BHK15; ATCC cell provider) cells, used for plaque assay and immunofluorescent focus assay (IFA), were cultured at 37°C in Minimum Essential Medium (MEM) supplemented with L-glutamine, non-essential amino acids (NEAA), and 10% heat-inactivated fetal calf serum (FCS) with 5% CO_2_. Green African monkey kidney (Vero; ATCC cell provider) cells, used to create LGTV stock and for IFA to determine LGTV stock titer, were cultured at 37°C in MEM supplemented with L-glutamine, NEAA and 10% heat-inactivated FCS with 5% CO_2_. LGTV TP21 wildtype strain, passage 2 (obtained from A. Pletnev, NIH-NAID, Bethesda, MD [38]) stock was amplified in Vero cells (multiplicity of infection 0.01) [39] and grown as described above, except with 2.5% heat-inactivated FCS, up to passage 4 (p4) to provide a working stock for experimental infections. Serial IFAs were conducted in parallel as previously described [40] in 96-well cell culture plates to determine LGTV stock titers.

### Production of non-infectious LGTV

To create non-infectious LGTV (UV-LGTV), LGTV p4 stock medium was placed in 48 well cell culture plates and treated with UV radiation at a distance of 11 cm from a standard (12.4 watt) UV lamp in a biological safety cabinet (Nuaire Labgard ES, Plymouth, MN) for 30 second intervals over a five minute period. LGTV inactivation was confirmed by blind passage of UV-LGTV on ~2 x 10^7^ ISE6 cells and ~80% confluent BHK15 cells, followed by immunofluorescent and plaque assay as described by Perera et al. [41] to demonstrate lack of infectivity.

### LGTV infection of ISE6 cells and measurement of infectious LGTV

IFAs were used to assess the level of LGTV infection in ISE6 cell populations. Detection of the LGTV non-structural protein 3 (NS3) was performed using YP-conjugated chicken anti-LGTV NS3 (provided by S. Best, NIH-NAID, Hamilton, MT) as primary antibody and IgG-conjugated goat anti-chicken, Alexa Fluor 488 (Invitrogen, Grand Island, NY; A11039) as secondary antibody. Cell nuclei were labeled with 4',6-diamidino-2-phenylindole (DAPI; Life Technologies, Grand Island, NY; D1306). Glass coverslips were used to culture and infect cells for the IFAs and were placed onto microscope slides, which were viewed on an Olympus model IX81F-3 microscope and images were collected using an Olympus U-CMAD3 camera. Fluorescence excitation was provided by the EXFO X-Cite Series 120PC and Olympus IX2-UCB. Image overlays were produced with Metamorph Basic v7.6.5.0 software.

To establish an MOI and time-point corresponding to optimal LGTV replication in ISE6 cells, three concentrations (MOIs of 7, 13, and 26) of LGTV were used to infect cells. For each, cells were fixed at 3, 9, 24, and 48 hpi with five technical replicates that were imaged under 20x magnification. On the basis of complete infection (>96%) of ISE6 cell populations between two MOIs (7 and 13) and time points (24 and 48), an MOI of 10 was selected for subsequent experiments for maximum infection. Separately, an assessment of the cumulative virus release was carried out in LGTV-infected ISE6 cells at a MOI of 10. Medium from these LGTV-infected ISE6 cells was harvested at 12 hour intervals for up to 120 hours, and subjected to plaque assays to measure replication.

### Preparation of peptide samples and mass spectrometry analyses

#### Preparation of ISE6 cell samples for LC-MS/MS analysis

To establish LGTV infection of ISE6 cells for LC-MS/MS analyses, ~2 x 10^6^ ISE6 cells in T25 flasks were adsorbed with LGTV p4 at an MOI of 10 for one hour as previously mentioned. Infection of >96% of the cell population was confirmed by IFA. In parallel, ISE6 cell populations were adsorbed with UV-inactivated LGTV and conditioned medium (obtained from uninfected Vero cell culture). The production of these three treatment groups representing cells treated with (a) infectious LGTV (LGTV), (b) non-infectious LGTV (UV-LGTV) and (c) conditioned medium only (mock control) each was replicated five times (five separate T25 flasks; n = 5 biological replicates). Samples (15 T25 flasks total) were harvested at 36 hpi and were adjusted to ~1.7 x 10^5^ cells to ensure an equal concentration of cells per replicate. Cells were then pelleted at 1,510 g for 5 minutes, culture supernatant was removed, and pellets were stored at -80°C. Cell pellets were thawed, re-suspended with hypotonic 100 mM ammonium bicarbonate buffer, and subjected to passive lysis (30 minutes) and mixed manually by pipetting at room temperature (RT). Protein concentration was determined using a NanoDrop 2000c (Thermo Scientific) in protein a280 mode (v1.2.1). Chloroform:methanol (2:1) extraction was performed and proteins from the aqueous phase was collected [42, 43]. Proteins were precipitated by addition of ice cold 100% acetone to samples, followed by vortexing for five seconds, and incubation at -20°C for one hour. Samples were spun at 16,000 g for five minutes and the protein pellet was re-suspended in 8M Urea supplemented with 10mM DTT and incubated at 37°C for 1.5 hours. Proteins were denatured by addition of a 50mM ammonium bicarbonate solution supplemented with acetonitrile (ACN), triethylphosphine (TEP), and 2-iodoethanol (97.5%:0.5%:2%) [42, 43]. Protein pellets were dried on a speed vacuum for two hours at 37°C and digested for 18 hours at 37°C in 50 mM trypsin (Sigma-Aldrich; T6567) solution (diluted in ammonium bicarbonate) at a ratio of 1:50 w/w trypsin:protein [43].

#### LC-MS/MS analyses and identification of proteins in ISE6 cell samples

Molecular species of trypsin-digested peptides were separated on a nanoLC system (1100 Series LC, Agilent Technologies, Santa Clara, CA). Peptides were loaded on an Agilent 300SB-C18 enrichment column for concentration and the column was switched into the nano-flow path for five minutes. Peptides were separated with a C18 reversed phase ZORBAX 300SB-C18 analytical column (0.75 μm x 150 mm, 3.5 μm) from Agilent.

The column was connected to the emission tip from New Objective and coupled to the nano-electrospray ionization (ESI) source of the hybrid ion trap mass spectrometer LTQ-Orbitrap LX (Thermo Scientific). The peptides were eluted from the column using an acetonitrile/0.1% formic acid (FA; mobile phase B) linear gradient. Specifically, the column was equilibrated with 95% H_2_O/0.1% FA (mobile phase A) for 5 min and proteins were eluted using a linear gradient of 5%-35% B for 50 minutes at 0.3 μL/min, followed by a linear gradient of 35%-100% B for 10 minutes. The column was washed with 100% of ACN/0.1% FA and equilibrated with 95% of H_2_O/0.1% FA before injection of the subsequent sample. A blank injection run between every five samples (of each treatment group) was completed to avoid carryover.

Operation was completed in the data-dependent positive acquisition mode in which full MS scan (resolution 30,000) was followed by four MS/MS scans. The four most abundant molecular ions were selected and fragmented by collision-induced dissociation (CID) using a normalized collision energy of 35%. Raw data were collected via Xcaliber (v 2.0.7). Database searches were conducted using Spectrum Mill (MS Proteomics Workbench v.03.02 software; Agilent Technologies). To identify peptides, a homology search was conducted against the VectorBase *I*. *scapularis* IscaW1.2 annotation [44] protein dataset (precursor mass tolerance = 0.05 Da; fragment mass tolerance = 0.6 Da; maximum of two tryptic mis-cleavages) using Spectrum Mill [42, 43]. Only those peptides with a Spectrum Mill score of ≥ 5 and scored peak intensity (% spi) of ≥ 70% were considered [42, 43, 45].

To account for false positives, a decoy (reversed) database search was performed using the MS/MS search option in Spectrum Mill. Peptide scores were compared to those of reversed peptide scores to obtain a delta forward-reverse score. The cut-off was established as >1 for +1 parent charged peptides and +2 parent charged peptides and >2 for +3 (and greater) parent charged peptides [45].

#### LC-MS differential analysis of LGTV-infected ISE6 cell samples

The Omics Discovery Pipeline (ODP; omicsdp.org) was employed for differential protein analyses with mass spectrometry proteomics [42, 43, 46, 47]. Raw data from LC-MS/MS runs (from 15 separate samples; 5 replicates for each of the three treatment groups) were uploaded and total ion chromatograph visualization was completed with each sample run of each treatment group. Deconvolution of spectra into peaks was completed for each sample utilizing XMass using the GISTool [48]. Alignment of peaks using XAlign [49] was conducted and normalization was accomplished from a number of different methods [50–52] that best fit the data. Normalized files were subject to statistical analyses and pattern recognition analysis. This heat map was created using the Heatmap.2 function of the ‘gplots’ package in R. For more information regarding the specific components of the ODP, refer to [46].

#### Statistical analyses

Following alignment and normalization of MS peaks, standard two-sample t-test was used to compare significant differences between averaged mass profiles of the treatment group samples (5 biological replicates) in an inter dependent fashion with a two-group analysis. An ANOVA was also utilized to identify significant masses across the averaged treatment group samples in an inter dependent fashion with a three-group analysis. Statistics were incorporated using the R statistical package (http://www.r-project.org/). Application of a false discovery rate based correction method [53] was performed with the significance tests.

The following methodology was used to account for the average fold change of a protein with multiple peptides: (1) Log of each peptide intensity value followed by (2) averaging these Log values, identified an averaged Log-valued protein intensity fold change; (3) the natural Log (ln) of the averaged Log-valued protein intensity fold change was determined in order to convert values to the original average fold change value for the corresponding protein. The “flattening” of the data was a stringent approach to identify the differential change in MS peak intensities for a particular protein. Fold change of >2 was considered an increase in expression, 0.5–2 fold change denotes no change in expression, and fold change of <0.5 corresponds to a decrease in expression.

### Assignment of ISE6 proteins to function, class, and pathway

Peptides with homology to *I*. *scapularis*, IscaW1.2 gene models were assigned to putative functional class by searching accession numbers against the KEGG orthology database (http://www.genome.jp/kegg/ko.html) and the KEGG pathway database (http://www.genome.jp/kegg/pathway.html). ISE6 proteins with orthology to KEGG entries were populated within KEGG pathways that also included mammalian and arthropod orthologs.

### Measurement of ISE6 cell concentrations

The concentration of cells in each sample (cells/ml) was estimated by counting cell number on a Scepter 2.0 Automated Cell Counter with 40 μM Scepter sensors (EMD Millipore; PHCC20040) in order to equalize cell numbers between biological replicates and between treatment groups prior to protein extraction. For cell population and growth analyses, initial cell counts (cells/mL) were determined manually using a hemocytometer and subsequently verified by sample analysis on the Scepter 2.0 Automated Cell Counter.

### Compound, cell viability, cell death assays

Trichostatin A (Sigma-Aldrich; T8552) and Oligomycin A (Sigma-Aldrich; 75351) were separately re-suspended in DMSO to a final concentration of 10 mM. 96-well plates, pre-treated with 0.01% Poly-L-Lysine (Sigma Aldrich; P4832), were separately seeded with ISE6 and Vero cells and incubated for 24 hours to final cell density of ~1 x10^5^ cells/96 well. ISE6 and Vero cells were infected with LGTV (passage 4, MOI of 10) and (passage 4, MOI of 3), respectively. Following adsorption, compounds diluted in DMSO, were added to cells to a final concentration of 0.01, 0.1, 1, and 10 μM (1% of total overlay medium) and cells were incubated at 37°C. Culture supernatant was collected at 36 hpi and used to quantify LGTV replication by plaque assay. To assess cell viability, cells were treated with alamarBlue reagent (AbD Serotec; BUF012A) diluted 1:10 with fresh medium for 12 and 2 hours, respectively. Fluorescence (excitation at 560nm, emission at 590 nm) was measured at 48 and 38 hpi using a Molecular Devices SpectraMax M5 plate reader coupled with SoftMax Pro v4.8 software. Control was solvent only. Five technical replicates were performed for each concentration with biological replicates (n = 2).

Trypan blue cell exclusion assay was used to assess mortality of ISE6 cells following LGTV infection. Poly-L-Lysine-treated 96-well plates were seeded with ISE6 cells for 48 hours to a cell density of ~9 x 10^4^ cells/well. Cells were treated with LGTV infection (MOI 10; p4 LGTV stock) or condition medium as described above. Cells were harvested at 12, 24, 36, and 48 hpi, centrifuged at 1,510 g for 5 min, medium was removed and the cell pellet was re-suspended in 1X PBS. Subsequently, a 1:1 0.4% trypan blue:cell suspension, was prepared, incubated for ~3 min at RT, the cells were immediately counted using a hemocytometer [54] and the percentage of stained ISE6 cells was determined for LGTV and mock treatments. Three technical replicates were collected per treatment with two biological replicates (n = 2).

## Results

### Characterization of LGTV growth in ISE6 cells

**Fig 1:**
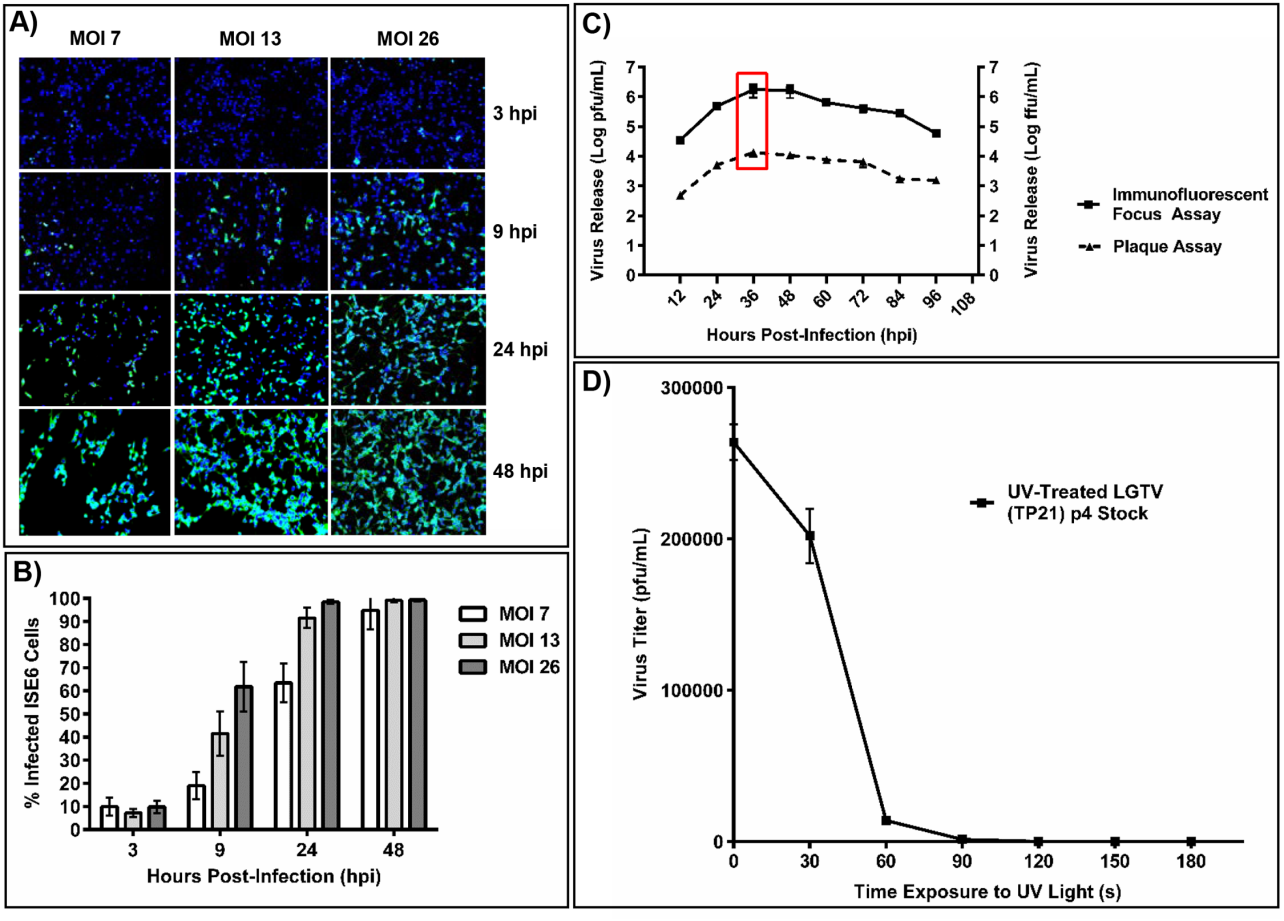
Quantification of LGTV infection in *I*. *scapularis* ISE6 cells via immunofluorescence and plaque assay. (A) Immunofluorescent detection of virus in ISE6 cells at 3, 9, 24 and 48 hours post infection (hpi) with LGTV MOIs of 7, 13 and 26. LGTV NS3 nonstructural protein (green), DAPI-stained nuclei (blue). (B) Percentage of infected ISE6 cells at 3, 9, 24 and 48 hpi following treatment with LGTV at MOIs of 7, 13 and 26 as determined from quantifying ISE6 cells with immunofluorescent LGTV NS3 expression. (C) Timecourse experiment showing amount of infectious LGTV (Log pfu/mL) released from ISE6 cells initially infected with LGTV MOI = 10 (n = 3). Titration was performed using both plaque assays and immunofluorescent focus forming assays in BHK15 cells. The red box corresponds to the time of peak release of infectious virus. (D) Plaque assays in BHK15 cells showing the reduction in infectious viral titer (pfu/mL) following treatment with UV-irradiated LGTV viral stocks for up to 300 seconds (n = 2).

IFA and plaque assays were used in time course experiments to assess levels of LGTV in ISE6 cells and to confirm UV inactivation of LGTV (Fig 1). Under the assay conditions described herein, IFA revealed that the maximum level of LGTV infection of the ISE6 cell population (>96%) corresponded to an MOI of 10 as determined by percentage of cells labeled with the LGTV NS3 protein (Fig 1A and 1B), and plaque assays revealed that the peak of LGTV release from ISE6 cells occurred at 36 hpi (Fig 1C). These conditions were selected for subsequent proteomic analyses. Plaque assays revealed that UV radiation for ≥120 sec was sufficient to achieve 100% inactivation of LGTV as determined by the lack of plaque formation (Fig 1D). The minimum time required for lack of plaque formation was 3.5 minutes. UV-LGTV used for proteomic analyses and subsequent assays was inactivated for five minutes.

ISE6 cell viability was reduced during the acute stage of infection with LGTV (i.e., ≤48 hpi) as measured based on presence of cellular reducing agents (FMNH_2_, FADH_2_, NADH, NADPH, and cytochromes). No change in cell growth or mortality was observed, as measured by counting cell population numbers and utilizing the trypan blue cell exclusion assay for LGTV-infected and mock-treated groups (S1 Fig).

### Effects of LGTV infection on the ISE6 proteome

Completion of the virus lifecycle as determined by release of infectious virus particles (Fig 1C) was observed in ISE6 cells infected with LGTV. In comparison, in cells treated with UV-inactivated virus (UV-LGTV) we observed no release of infectious virus particles (Fig 1D). Comparative proteomics analyses were used to identify proteins expressed throughout the process of cell infection (LGTV) versus those associated only with viral attachment and entry of the host cell (UV-LGTV). The sequence of proteomic analyses performed using the three treatments (LGTV, UV-LGTV, and mock) is shown in S2 Fig and S1 Table.

**Fig 2:**
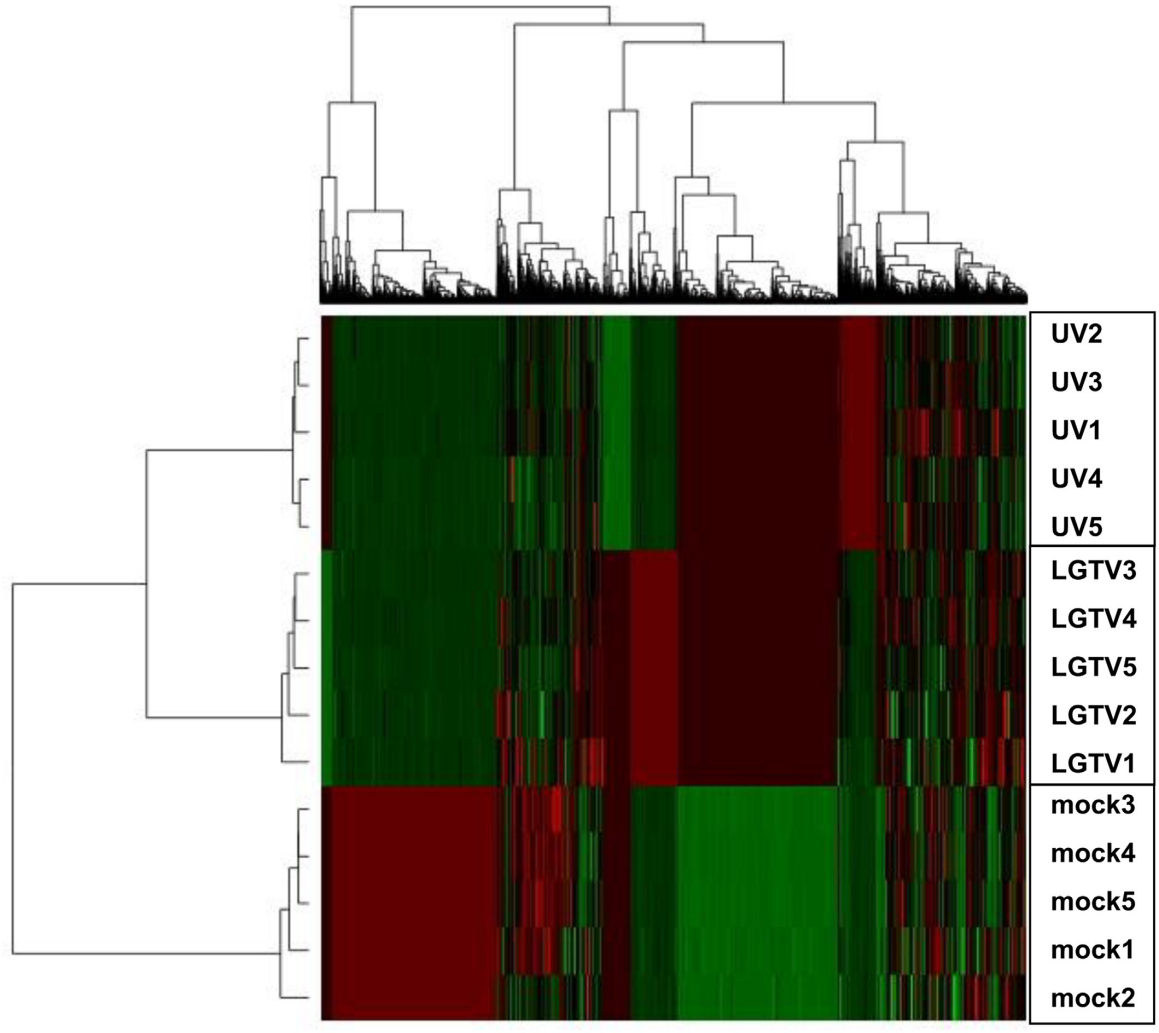
Hierarchical clustering of MS peak profiles of ISE6 cells treated with LGTV, UV-LGTV, and mock. M1-5, mock1-5 samples; LGTV1-5, LGTV-infected samples 1–5, and UV-LGTV1-5, UV-LGTV samples 1–5. Vertical rows depict n = 5 biological replicates. Horizontal rows correspond to significant MS peaks of peptides/proteins at 36 hours post infection/treatment. Clustering analysis shows common patterns of protein expression profiles shared between the three treatment groups. The red-green color scale denotes the Z score fold change with red representing a Z score of -2 and green denoting a Z score of 2 [41].

**Fig 3:**
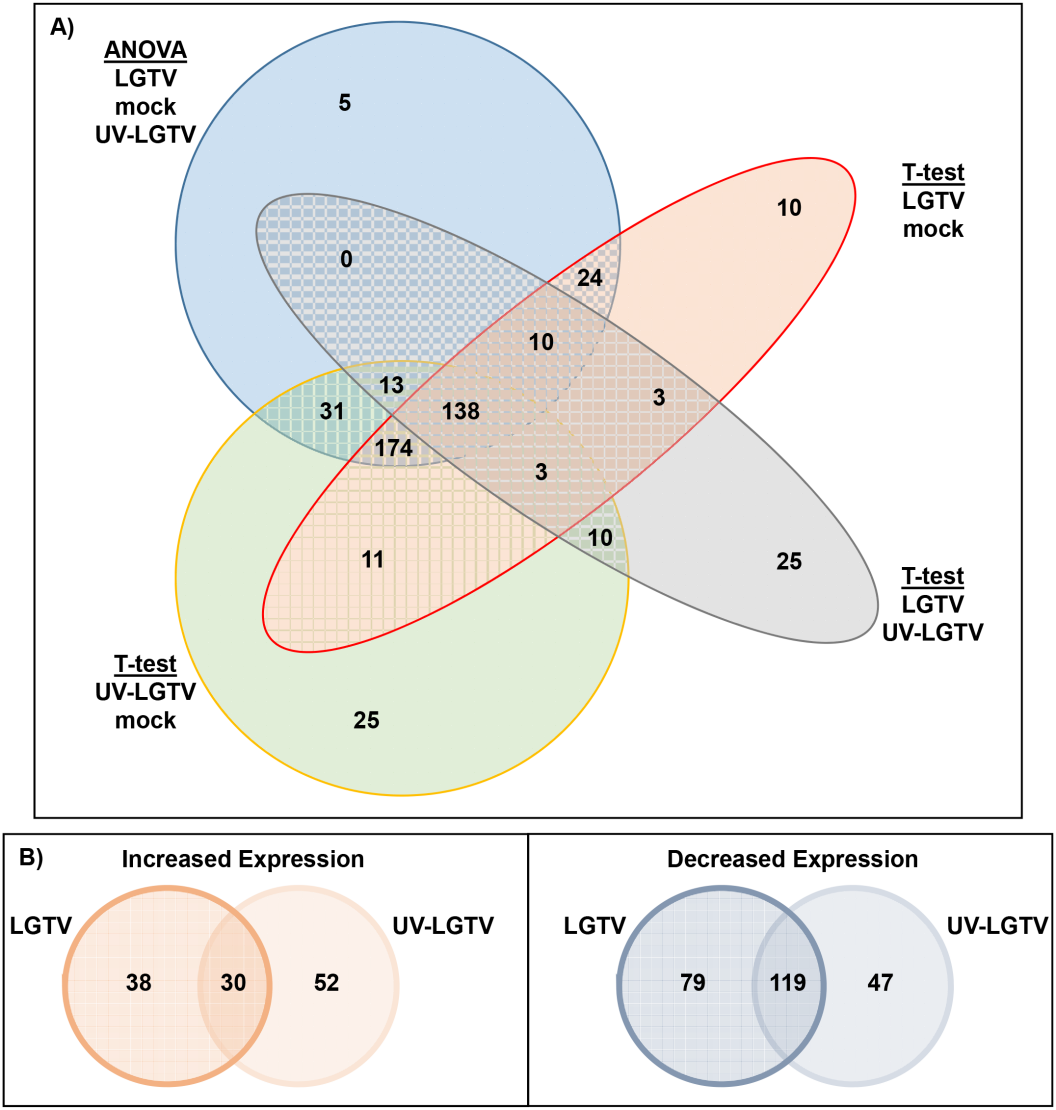
Identification and expression of ISE6 cell proteins following treatment to LGTV, UV-LGTV, and mock. (A) Four statistical analyses were performed using the ODP output. This included a three-way ANOVA of treatment groups LGTV, UV-LGTV, mock and two-way t-test comparing the LGTV vs. mock, UV-LGTV vs. mock, and LGTV vs. UV-LGTV samples. The Venn diagram shows unique and common ISE6 protein identifications from these datasets. (B) Venn diagrams showing the numbers of ISE6 proteins identified as exhibiting increased or decreased expression following treatment to LGTV or UV-LGTV unique or common to sample groups (see S2 and S5 Tables for the complete list of proteins identified in the analyses).

**Fig 4:**
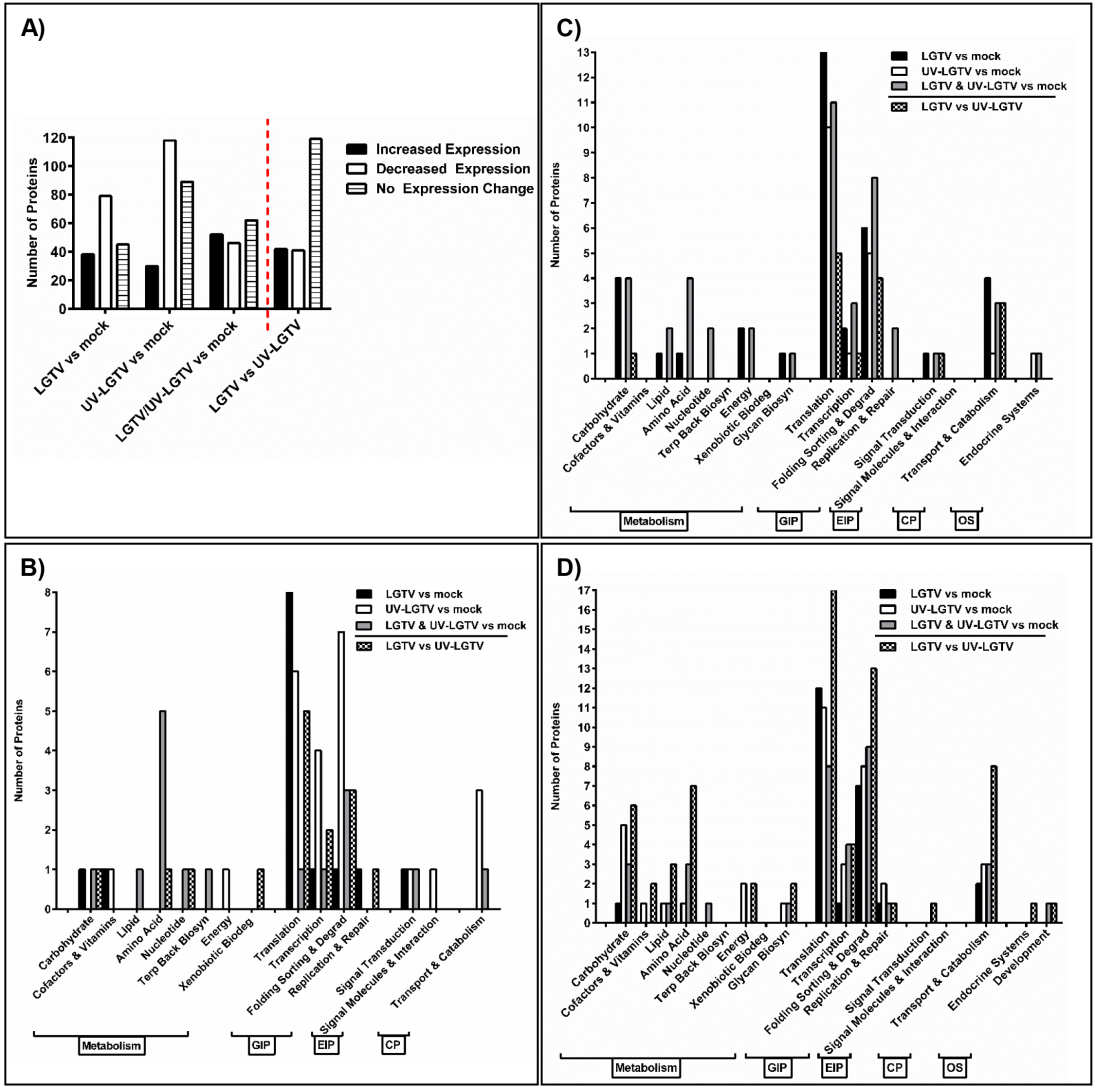
ISE6 proteins identified in KEGG pathways with differential expression following LGTV, UV-LGTV and mock treatment. (A) Total number of ISE6 cell proteins categorized by treatment and change in expression (increase/decrease/no change). Total number of proteins showing (B) increased expression, (C) decreased expression, and (D) no change in expression following LGTV infection and UV-LGTV treatment as compared to mock-treated cells and in LGTV-infected cells as compared to UV-LGTV-treated cells. Proteins were categorized by the KEGG classes for cellular function: metabolism, genetic information processing (GIP), environmental information processing (EIP), cellular processes (CP), and organismal systems (OS).

LC-MS data were compared for LGTV, UV-LGTV and mock samples (Fig 2). The expression pattern of LC-MS peaks for LGTV samples was more similar to that of UV-LGTV samples than to that of mock samples. The t-test and ANOVA (four separate statistical analyses) were used to identify proteins that exhibited differential expression (p < 0.05) between LGTV and UV-LGTV samples as compared to the mock samples (Fig 3A). In total, 486 ISE6 proteins (S2 Table) were identified based on homology to NCBI/VectorBase accessions. Of these, 266 and 248 proteins were identified as differentially expressed in the LGTV and UV-LGTV samples, respectively compared to mock samples. Sixty-eight proteins had increased expression, while 198 proteins showed decreased expression in the LGTV samples as compared to mock samples. Additionally, 82 and 166 proteins showed increased and decreased expression (Fig 3B), respectively in the UV-LGTV samples in comparison to mock samples. Overall, 243 proteins (50%) exhibited decreased expression while 120 (24.7%) showed increased expression in LGTV and UV-LGTV samples as compared to the mock treatment (Fig 4A).

### Functional analyses of *I*. *scapularis* ISE6 proteins

Of the 486 ISE6 proteins identified in this study, 265 (54.5%) mapped to orthologous proteins in the KEGG database, while 221 proteins had no match (KEGG; genome.jp/keg/ko). Of the 265 proteins, 176 (36.2%) mapped to 66 KEGG pathways and 16 KEGG modules (S3A Fig and S3 Table). The KEGG pathways identified in the present study were categorized into five cellular functions: “metabolism”, “genetic information processing”, “environmental information processing”, “cellular processes”, and “organismal systems”. The majority of proteins (52%) were identified to the functional category “genetic information processing”, followed by “metabolic” (38.7%) and “cellular” (6.3%), “environmental information processing” (2%), and “organismal systems” (1%) (S3B Fig).

LGTV samples exhibited the highest number of proteins (53) identified to the KEGG pathway “genetic information processing” (Fig 4B–4D). Within this group, eight proteins exhibited increased expression and were classified in the pathway, “translation” (Fig 4B). For UV-LGTV samples, the majority of ISE6 proteins (57) were also classified in the pathway, “genetic information processing”. The majority of proteins exhibiting increased expression (17) were classified in the protein processing pathways of “folding, sorting, and degradation” (7 proteins; 41.2%), followed by “translation” (6 proteins; 35.3%) and “transcription” (4 proteins; 23.5%).

Proteins from the LGTV and UV-LGTV samples that lacked a match to KEGG database entries, also displayed differential expression. Of these, 30 proteins had increased expression and 91 had decreased expression in LGTV samples in comparison to mock samples (S4 Fig and S2 Table). Additionally, 38 and 85 proteins were identified with increased and decreased expression, respectively, in the UV-LGTV samples as compared to mock samples.

### Changes in the ISE6 cell proteome following LGTV infection

**Fig 5:**
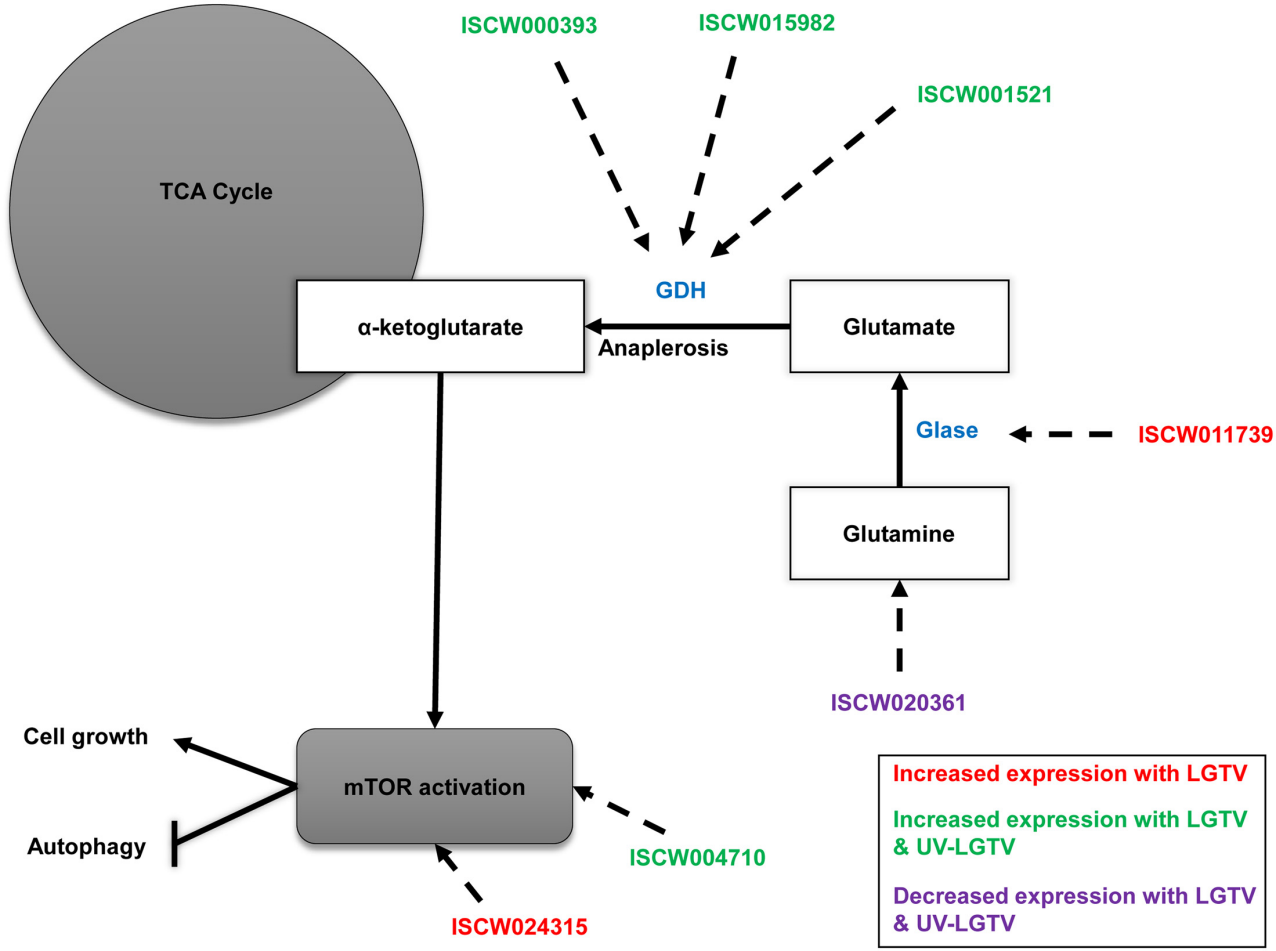
ISE6 proteins associated with the TCA cycle and glutaminolysis. ISE6 glutaminolysis and mTOR signaling proteins altered with LGTV infection are shown. GDH denotes glutamate dehydrogenase enzymes and Glase denotes glutaminase enzymes. ISCWxxxxxx denotes corresponding VB accession ID for corresponding *I*. *scapularis* protein.

**Fig 6:**
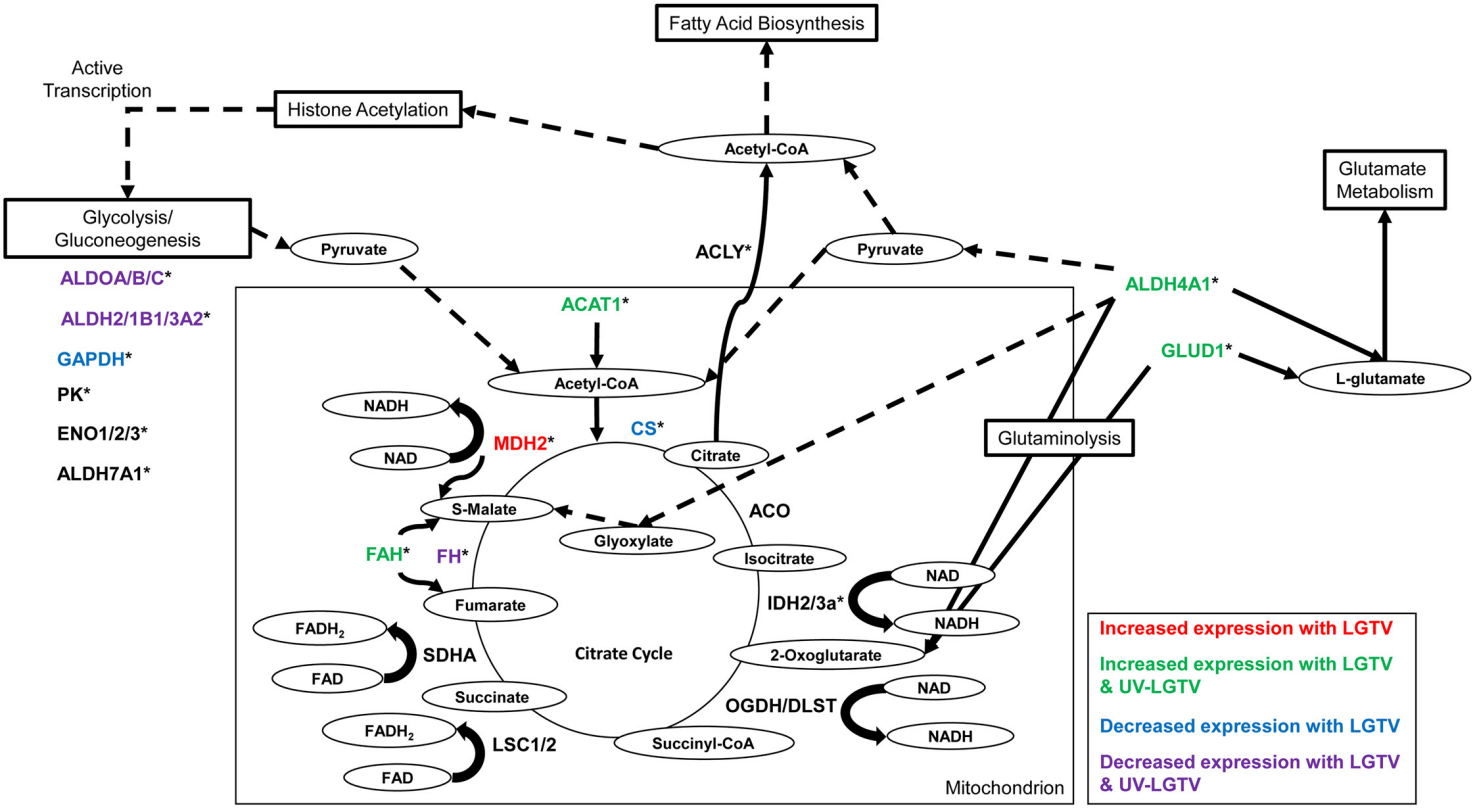
Citrate cycle showing ISE6 proteins that exhibited increased/decreased expression following treatment to LGTV and UV-LGTV. The enzymes are indicated with KEGG abbreviated nomenclature and the corresponding substrates are shown in circles. * denotes proteins identified in this study. Dotted lines denote indirect involvement with production. The increased expression of malate dehydrogenase (MDH2) is unique to LGTV-treated cells while increase in the expression of acetyl-CoA acetyltransferase 1 (ACAT1), delta-1-pyrroline-5-carboxylate dehydrogenase (ALDH4A1), glutamate dehydrogenase (GLUD1), and fumarylacetoacetase (FAH) is common to cell samples following LGTV infection and UV-LGTV treatment. Decreased expression of citrate synthase (CS) and glyceraldehyde-3-phosphate dehydrogenase (GAPDH) was observed in LGTV-treated cells and decreased expression of fumarate hydratase (FH), aldolase A/B/C fructose-bisphosphate (ALDOA/B/C), and aldehyde dehydrogenase 2/1B1/3A2 family protein (ALDH2/1B1/3A2) was observed in both LGTV-infected and UV-LGTV—treated cells. ATP citrate lyase (ACLY), aconitase (ACO), isocitrate dehydrogenase 2/3a (IDH2/3a), oxoglutarate/alpha-ketoglutarate dehydrogenase complex (OGDH/DLST), succinyl-CoA synthetase alpha/beta subunit (LSC1/2), succinate dehydrogenase flavoprotein subunit (SDHA), pyruvate kinase (PK), enolase 1/2/3 (ENO1/2/3), and aldehyde dehydrogenase 7A1 family protein (ALDH7A1).

Proteins that showed an increase in expression in LGTV samples were mapped onto the KEGG functional categories of cell signaling (CYC, STK3, RPS6), proteolysis (UCHL3, PSMA, UBE2N), carbon-nitrogen hydrolase activity (DDAH, VNN), replication and mRNA processing (PARP, TRA2, CUTL, H2A, CSTF2), translation (RPS6, RPL17, AARS, NARS), glutamate metabolism/glutaminolysis (prostate-specific transglutaminase, putative ISCW011739; Fig 5 and S4 Table), pyruvate metabolism and energy association (MDH2; Fig 6). Proteins that exhibited decreased expression were associated with the functional categories of glycolysis (GAPDH; Fig 6), energy processes (ATP5H, ATP5A1), and mRNA surveillance (PABPC, PELO, MSI, THOC4).

### Changes in the ISE6 cell proteome following UV-LGTV treatment

Proteins exhibiting increased expression in UV-LGTV samples were mapped onto the KEGG functional categories of signaling (RHOGDI, RAB35, SIP, LAMC1), cytoskeletal components, (ACTN, TUBA), unfolded protein response and ER-associated degradation (HSPA1_8, RAB7A), lysosomal functions (PSAP), and phagosome functions (RAB7A). Proteins that exhibited a decrease in expression were associated with transport (BAP31), cell survival (BAP31, HYOU1, DERL1, GROEL), cell growth (SUMO, NOP10, MAD1L), translation (NOP10), and protein folding (GROEL).

### Changes in the ISE6 cell proteome in common to both LGTV infection and UV-LGTV treatment

Responses common to LGTV and UV-LGTV samples included proteins exhibiting increased expression and associated with signaling (ITGB, MO25), cytoskeletal structure perturbation (TLN), amino acid metabolism (ACAT, DP5CD, GLUD1, CARP, FAH), glutamate metabolism/glutaminolysis (DP5CD, GLUD1, membrane protein, putative ISCW001521; Fig 5 and S4 Table), RNA interference (AUB), and energy-production (ACAT). Proteins with decreased expression and common to both treatment groups were classified to KEGG functions of glycolysis (ALDOA/B/C, ALDH2/1B1/3A2; Fig 6), energy association (ATP5D, ATP5B), RNA interference (VIP), and structural manipulation (ACTB_G1, TUBB).

### Comparative proteomics of tick, mosquito and human-flaviviral infection

**Fig 7:**
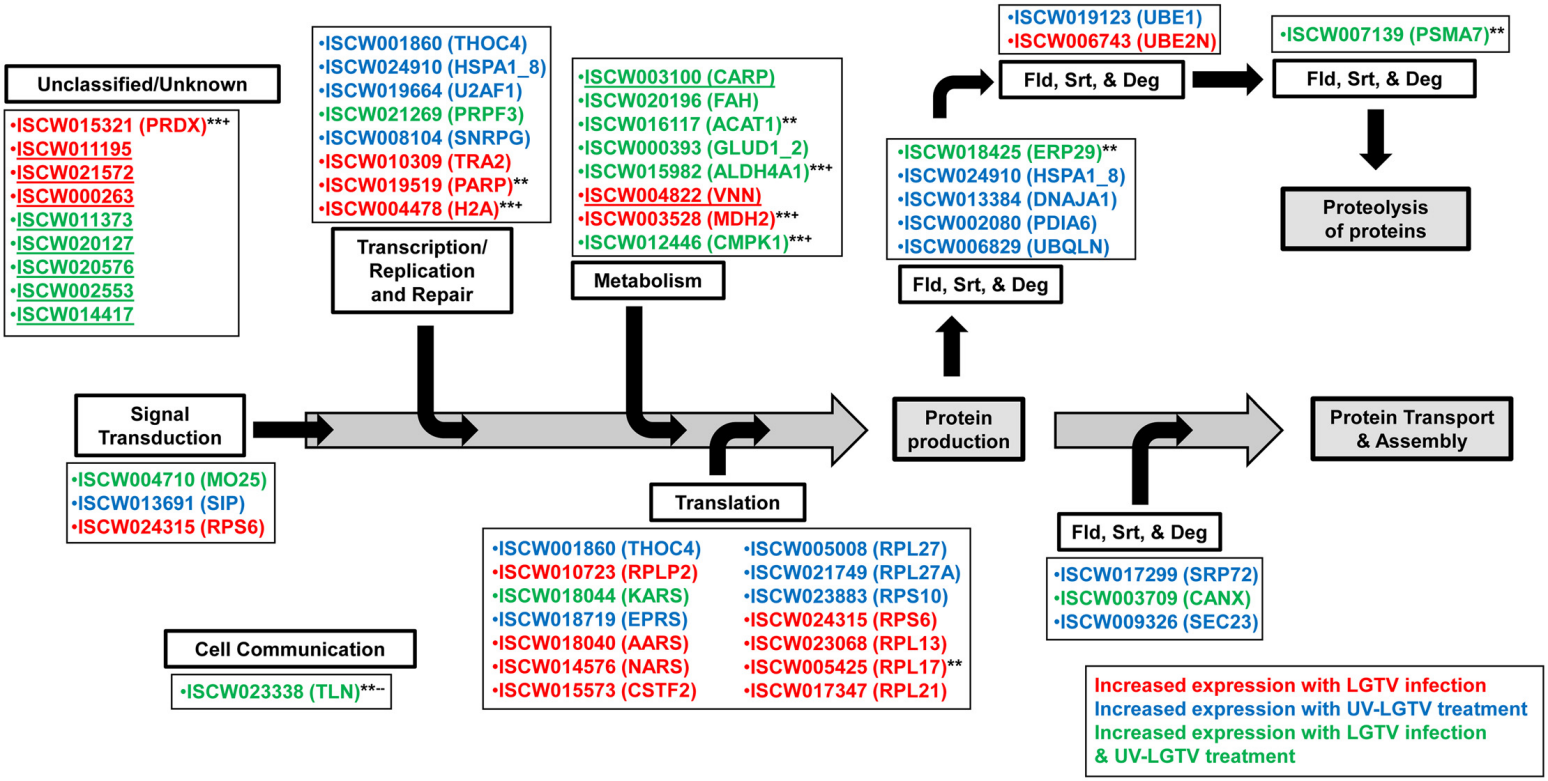
ISE6 proteins with increased expression associate with pathways for protein production, transport, assembly, and proteolysis. The ISCW accession numbers corresponding to proteins identified with increased expression in LGTV-infected and UV-LGTV-treated cells are shown (KEGG abbreviated nomenclature provided as well if available in parentheses). The schematic presents potential mechanisms for LGTV-induced perturbation and increase in cell protein expression. See S5 Table for information on proteins and KEGG protein classes. Underlined proteins denote ISE6 proteins exhibiting increased expression following flavivirus infection not identified before. “Fld, Srt, & Deg” is the folding, sorting, and degradation KEGG pathway. ** denotes ortholog in human [19]; + denotes human ortholog showing increased expression following HCV infection;—denotes human ortholog showing decreased expression following HCV infection. Gray boxes denote cellular protein functions. White boxes denote KEGG pathways.

185 of the 265 ISE6 proteins with orthology to KEGG entries (70%) were also identified in a proteomics study of HCV infection of HUH7.5 cells [19] (S5 Fig). Sixteen ISE6 proteins (6%) matched orthologs identified in a study of West Nile virus (WNV) infection of Vero cells [55], 16 proteins matched orthologs in a yeast two-hybrid study of flavivirus-host interactions [56], and 15 proteins (5%) matched orthologs identified in *Aedes aegypti* infected with dengue virus (DENV) [28]. A subset of proteins that exhibited increased expression following LGTV infection and/or UV-LGTV treatment and matched proteins in the studies above, were associated with protein synthesis and proteolysis (Fig 7 and S5 Table). Of the remaining 66 proteins (24.9%), those that exhibited increased expression in LGTV samples were classified in the KEGG functional categories of proteolysis (PMSA, CARP), ATP association/interaction (PSMA, ANMK), cell and matrix adhesion (VNN, ITGB), and as well as oxidative stress and redox homeostasis (VNN and conserved hypothetical protein ISCW020127-PA). Additionally, the cellular function of hydrolase activity was suggested by increased expression of PSMA and VNN (S5 Table).

### Functional analyses of ISE6 proteins using small molecule *in vitro* assays

**Fig 8:**
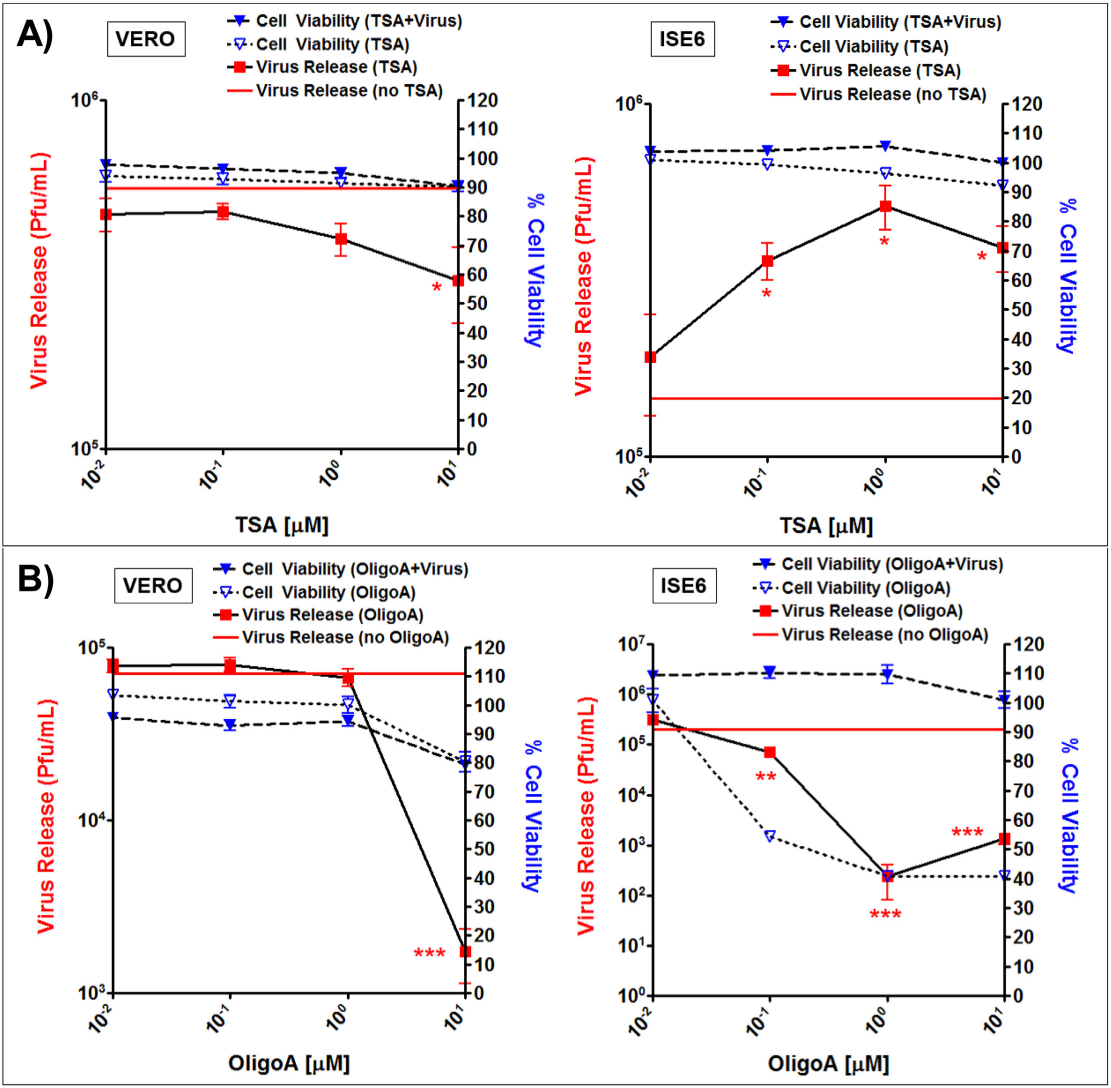
*In vitro* assays impact of (A) Trichostatin A and (B) Oligomycin A on LGTV replication. Trichostatin (TSA) and Oligomycin A (OligoA) concentrations on the x-axis, percent cell viability on the right y-axis, and virus release in pfu/mL on the left y-axis. Compound assays out of Vero cells are shown in the left panels and out of ISE6 cells are shown in the right panels. Release of LGTV was assessed by viral titer (pfu/ml) using plaque assays in BHK15 cells. Cell viability was determined with alamarBlue reagent and fluorescent assay and percentage was normalized against solvent only control in both LGTV-infected and mock-treated ISE6/Vero cells. * denotes p<0.05 and *** denotes p<0.001. Standard error shown in error bars with five technical replicates. Two biological replicate experiments were completed.

In order to manipulate metabolic functions and subsequent LGTV infection, small molecule assays were completed. In cellular assays, Trichostatin A (TSA), a compound known to inhibit histone deacetylase (HDAC) and to activate enzymes involved in intermediate metabolism, including MDH2, decreased viability of Vero cells (with and without LGTV infection) and LGTV replication (as measured by a decrease in release of infectious virus particles) at increasing concentrations (Fig 8A). Conversely, an increase in TSA concentration was associated with an increase in the viability of LGTV-infected ISE6 cells and an increase (~0.5 log pfu) in LGTV replication (Fig 8A). Oligomycin A (OligoA), a small molecule inhibitor of the mitochondrial H+ ATPase pump, known to inhibit terminal processes of the electron transport chain by reducing ATP production, was associated with a decrease in the viability of Vero cells (~20% reduction) and ISE6 cells (~60%) at increasing concentrations. Significant reduction of LGTV in the mammalian (~1.5 log reduction in pfu in Vero cells) and tick (~2 log reduction in pfu in ISE6 cells) system was observed with increasing concentrations of OligoA (Fig 8B).

## Discussion

We used an LC-MS/MS proteomics approach to analyze changes in the global protein expression profile of *I*. *scapularis* ISE6 cells following infection with LGTV and identified tick proteins tied to flavivirus infection and replication. The present study focused on proteins expressed during 36 hours post infection or the period of peak LGTV release from infected ISE6 cells (suggested 36–48 hpi in combination with published studies [57]). In total, 486 ISE6 proteins were identified, and of these, 66 exhibited increased expression and 198 proteins exhibited decreased expression following LGTV infection. Two hundred and sixty-five of the proteins identified (54.5%) had orthology to proteins of known function from a variety of eukaryotes (S1 and S3 Figs). Finally, we present *in vitro* small molecule data to demonstrate that metabolism in the mitochondria may be critical for tick-borne flavivirus infection.

### Impact of LGTV infection on signaling in ISE6 cells

Several proteins were identified were included in notch and mTOR signaling pathways. The putative histone deacetylase 1,2,3 (ISCW007830-PA) exhibited decreased expression in LGTV and UV-LGTV samples. Several studies [58, 59] suggest a link between herpesvirus infection and gene regulation through with the binding of viral proteins to histone deacetylases [59]. We hypothesize that LGTV infection may impact the regulation of ISE6 genes via effects on histone deacetylase. In other systems, it has been shown that histone deacetylase can act as a co-repressor in the notch signaling pathway. TSA traditionally binds and inhibits histone deacetylases and treatment of ISE6 cells with TSA during LGTV infection increased LGTV replication, suggesting that LGTV infection impacts gene regulation through histone deacetylases. The putative 40S ribosomal protein S6 (ISCW024315-PA) and Mo25 (ISCW004710-PA) exhibited increased expression in LGTV cells. These proteins are members of the mTOR signaling pathway which has been implicated in human cytomegalovirus (HCMV) infection of mammalian cells [60, 61] and DENV infection of *A*. *aegypti* mosquitoes [62]. Increased expression of Mo25 may reflect a cellular stress response while increased expression of S6 may reflect an increase in translation to maintain growth of the infected cell or facilitate LGTV replication. Manipulation of mTOR signaling has been noted with WNV infection in mammalian systems [63]. The putative calcyclin-binding protein CacyBP (ISCW013691-PA) known to function in the Wnt signaling pathway in other systems, had increased expression in UV-LGTV-treated cells and decreased expression in LGTV-infected ISE6 samples. Our observation suggests an increase in proteolysis following virus treatment since the Wnt pathway is associated with the Ca^2+^-dependent, ubiquitin-mediated proteolysis pathway. Future investigations regarding roles of post-translational modifications in regulating signaling pathways following tick-borne flavivirus infection is necessary.

### ISE6 anti-viral responses perturbed by LGTV infection

Recently, the piwi-interacting RNA (piRNA) pathway has been implicated in the antiviral response of mosquitoes [64] and tick *I*. *scapularis* IDE8 cells [65]. Esther et al. 2014 identified three paralogs (ISCW015916, ISCW0021130, and ISCW011768) of the tick *I*. *scapularis* argonaute (aubergine) protein as antiviral factors to LGTV infection. The *I*. *scapularis* aubergine protein possesses the paz and piwi domains [66] associated with RNA binding. Homologs of these proteins were not identified in this study, although a homolog of argonaute (AUB; ISCW011373-PA) was identified that exhibited increased expression in both LGTV and UV-LGTV ISE6 cells and may play an antiviral role in LGTV infection.

Histone (H2A) is involved in DNA binding and chromatin packing of DNA, and therefore likely has a role in gene regulation and downstream host protein translation that may be important for homeostasis. The *I*. *scapularis* H2A (ISCW004478-PA) exhibited increased expression in LGTV-infected ISE6 cells. H2A also had increased expression during DENV infection of HUH7 liver cells and binds with the capsid protein to inhibit nucleosome formation in these human cells [67]. This protein has also been found to bind antisense RNA [68], also suggesting a possible anti-pathogen role as a result of changes in gene regulation.

The proteasome subunit alpha type protein (ISCW021572-PA) exhibited increased expression in LGTV samples and the 20S proteasome, regulatory subunit alpha type PSMA7/PRE6 (ISCW007139-PA) had increased expression in both LGTV and UV-LGTV samples. These proteins are subunits of the proteasome-associated 20S core particle and may exert antiviral roles through proteolysis and transcriptional regulation. Protein subunits of the proteasome have been shown to play a role in HCV internal ribosome entry site (IRES)-mediated translation [69] and may also interact with the HIV protein TAT and HBV protein HBX [70, 71].

### Role of actin polymerization in ISE6 cells following LGTV infection

Decreased expression of actin was observed in both LGTV-infected and UV-LGTV-treated samples. Cofilin (CFN; ISCW006326-PA), a putative actin-depolymerizing factor, exhibited decreased expression in these samples. CFN was also identified in a proteomic study of HCV-infected HUH7.5 cells [19]. Actin polymerization is involved with formation of actin stress fibers, a process that may facilitate vacuole formation [72] and mammalian neuronal cell entry of Japanese encephalitis virus [73]. UV-LGTV-treated cells exhibited increased expression of the signaling and structural proteins RHOGDI, and ACTN and TUBA, respectively. RHOGDI has been implicated in actin depolarization [74] and showed increased expression in HCV-infected HUH7.5 cells [19] at an early (12 hpi) infection time point. ACTN showed increased expression in HUH7.5 cells at early (24 hpi) and intermediate (48 hpi) time points post HCV infection and increased expression in UV-HCV-treated cells at a late (72 hr) time point. In the present study, we observed increased expression of ACTN in UV-LGTV samples at the 36 hpi time point. In addition to crosslinking actin fibers and facilitating filament assembly, ACTN been shown to bind the HCV nonstructural proteins NS3 and NS5 [56, 75]. We hypothesize that this protein may assist LGTV cell entry in ISE6 cells.

### Maintenance of metabolism in ISE6 cells following LGTV infection

The proteins acetyl-CoA acetyltransferase (ACAT1; ISCW016117) and aldehyde dehydrogenase 4A1 (DP5CD; ISCW015982) exhibited increased expression in LGTV-infected cells. These enzymes operate upstream of the TCA cycle and are associated with the production of acetoacetyl-CoA and pyruvate, respectively during cellular metabolism (Fig 6). This result suggests an increase in acetyl-CoA production following viral infection. Interestingly, citrate synthase (CS; ISCW009586) showed decreased expression following LGTV infection and may reflect a reduction of TCA protein activity late in LGTV infection. We observed a decrease in expression of fumarate hydratase (FH; ISCW020593) that may also similarly reflect reduction of TCA protein activity late in LGTV infection. The increased expression of MDH2 (ISCW003528), a protein involved in the final steps of the TCA cycle, may produce an increase in oxaloacetate, S-malate, and NADH in ISE6 cells. Moreover, increased expression of fumarylacetoacetase (FAH; ISCW020196) may increase fumarate, also involved in the final steps of the TCA cycle. ACAT1, DP5CD, MDH2, and FAH may aid in maintaining the TCA cycle late in LGTV infection. In parallel, these observations suggest an impact of LGTV on the TCA cycle at 36 hours post infection that may be linked to successful replication of the virus.

Our observation of a decrease in expression of fructose-bisphosphate aldolase (ALDOA), glyceraldehyde-3-phosphate dehydrogenase (GADH), aldehyde dehydrogenase family 7 member A1 (ALDH3A2), and pyruvate kinase (PKLR) in LGTV-infected and UV-LGTV-treated cells, suggests an impact of LGTV on glycolytic processes. This finding is at odds with that of Patramool et al 2011, who observed that DENV-infected C6/36 *A*. *albopictus* cells [27] exhibit increased glycolysis. The *in vivo* study of Tchankouo-Nguetcheu et al 2010 highlighted an increased expression of glycolytic proteins in the midgut tissues of DENV-infected *A*. *aegypti* [28]. Diamond et al 2010 also identified members of the glycolysis pathway that exhibited increased expression at early to intermediate time points (i.e., prior to peak release of infectious virus) following HCV infection, but not at the late (during and following peak release of virus from the cell) time point [19]. Although ticks are exclusive blood feeders and mosquitoes regularly take sugar meals between blood meals, these data suggest the possible increase in glycolysis at early to intermediate time points post flaviviral infection, but a decrease in glycolysis at later time points.

### Identification of candidate protein targets associated with LGTV replication

Our *in vitro* studies have shown that the compounds TSA and OligoA can affect levels of LGTV replication, presumably through impacts on a variety of cellular metabolic processes. TSA is thought to inhibit histone deacetylases and stimulate the acetylation of histones and metabolic enzymes, while OligoA may inhibit oxidative phosphorylation and electron transport. OligoA may activate AMPK activity [76], inhibit ATP production, and affect cellular energy levels. Clearly, further studies are required to determine the mode of action of TSA and OligoA in the LGTV-ISE6 system.

Glutaminolysis can produce an alternative energy source for the cell by generating ATP during the conversion of glutamine to α-ketoglutarate. Although tick medium has relatively large amounts of glutamine, glutamic acid, and α-ketoglutarate, increased expression of proteins associated with glutaminolysis (Fig 5 and S4 Table) suggest that LGTV infection of ISE6 cells may stimulate glutaminolysis and the production of α-ketoglutarate, a key intermediate in the TCA cycle. Studies suggest that glutaminolysis is manipulated during infection of human cells by both HCMV [77, 78] and HCV [19]. Thus, glutaminolysis and α-ketoglutarate are likely critical not only for maintaining the TCA cycle, but also supporting oxidative phosphorylation and ATP production in the infected cell. Additionally, stimulation of α-ketoglutarate has been shown to increase mTOR activity [79, 80] which operates in parallel with glutaminolysis. In ongoing studies, we are assessing viral manipulation of glutamate dehydrogenase (GDH) activity using inhibitory compounds with the goal of disrupting flaviviral infection.

### Summary

To contribute to an improved understanding of flavivirus-*I*.*scapularis* interactions, we developed an *in vitro* system to identify changes in ISE6 protein expression following infection with the TBF, LGTV. We present the first study to identify ISE6 proteins that are differentially-expressed following LGTV infection. In total, 486 proteins were identified with 66/198 showing increased/decreased expression following LGTV infection and 82/166 showing increased/decreased expression following UV-LGTV treatment. We identified proteins associated with the cellular functions of genetic information processing (GIP), metabolism, cellular processes, environmental information processing, and organismal systems. The majority of proteins populate GIP-specific pathways followed by metabolism-specific pathways.

The identifications of these proteins provide a critical resource to improve understanding of the *I*. *scapularis* proteome, improve gene annotations, and facilitate further studies in the tick cell culture system. Further understanding of protein function can also be achieved using approaches such as IFA, targeted mass spectrometry, small molecule *in vitro* assays, and RNAi. The present study is an important first step toward identifying tick proteins tied to LGTV replication as candidates for anti-tick vaccines and/or as targets for therapeutic screening to disrupt tick-borne flavivirus transmission.

## Supporting Information

S1 Fig ISE6 cell viability, growth, and mortality following LGTV infection. Alteration of ISE6 cell viability (A) at 12–24, 24–36, 36–48 hours post infection are shown in parallel with cell growth/population numbers (B) at 24, 36, 48 hours post infection, and cell mortality percentage (C) at 12, 24, 36, 48 hours post infection with and without LGTV (mock-treated) infection. * denotes p<0.05 and ** denotes p<0.01. Standard error shown in error bars with five technical replicates. Two biological replicate experiments were completed.
(TIF)

S2 Fig Summary of proteomic analysis of LGTV-infected and UV-LGTV-treated *I*. *scapularis* ISE6 cells. After whole cell sample harvest of treated ISE6 cells, cell pellet samples were subject to lipid removal, protein precipitation, peptide denaturation, and tryptic digest of peptides. Samples were prepared for the separation phase (nano LC) by injection, using electrospray ionization (ESI). Mass analysis of the precursor ion spectra was completed, followed by the second fragment ion MS/MS dimension for downstream peptide identification. Two group and three group statistical analyses with ISE6 cells treated with virus (LGTV), UV-inactivated virus (UV-LGTV), and no virus (mock) were compared utilizing a proteomic/metabolite pipeline, Omics Discovery Pipeline (ODP). After identification of significantly-changing (p < 0.05) MS peaks from LGTV-infected and UV-LGTV-treated ISE6 cells, corresponding peptides were identified to specific *I*. *scapularis* proteins (VectorBase *I*. *scapularis* WIKEL genome IscaW1.2 predicted protein set database). ISE6 proteins were then subject to protein function and pathway analyses (via KEGG). See materials and methods section for more detail.
(TIF)

S3 Fig ISE6 protein orthology and cellular function distribution of proteins found in KEGG pathways and modules. (A) *I*. *scapularis* ISE6 proteins with KEGG-mapped orthologs (or KEGG orthology [KO]) help to identify cellular pathways in *I*. *scapularis* (genome.jp/kegg/ko). To be identified in a KEGG pathway, KO is required. ISE6 proteins with KO and not identified in *I*. *scapularis* (KEGG) pathways are also included. (B) Percent cellular function distribution of proteins found in the 66 identified *I*. *scapularis* (KEGG) pathways with 16 modules.
(TIF)

S4 Fig Summary of differentially-expressed ISE6 proteins without identified pathways. Expression of ISE6 proteins with (A) or without (B) orthology and no identified pathways. Refer to S2 Table for more specifics on the proteins. Red dotted line denotes differentially-expressed proteins in LGTV-infected ISE6 cells compared to UV-LGTV-treated ISE6 cells (no comparison to mock-treated ISE6 cells).
(TIF)

S5 Fig Number of ISE6 proteins corresponding to orthologous proteins identified in proteomic analyses of flavivirus-host systems. Corresponding percentages correspond to the number of tick ISE6 orthologs identified with orthologs identified in: ^α^ S5 Fig, S7 Fig, and S11 Fig of Khadka et al. [56]; ^μ^S2 Table of Tchankouo-Nguetcheu et al. [28]; ^β^Tables 1 and 2 of Pastorino et al.[55]; ^Δ^S1 Table of Diamond et al.[19].
(TIF)

S1 Table Summary of analyses used to identify proteins from LGTV-infected and UV-LGTV-treated ISE6 cell samples. (DOCX)

S2 Table 486 significant, *I*. *scapularis* ISE6 proteins identified. The total number of *I*. *scapularis* ISE6 proteins is based off of ≥1 peptide identification and ≥1 statistical analysis (p < 0.05) identification (four total analyses). From S1 Table, the filter process in detail is listed and Fig 2 is a pattern representation including the 486 proteins listed in S1 Table. Fold change of >2 corresponds to an increase expression, 0.5≤fold change≤2 denotes no change in expression, and fold change of <0.5 correlates with decreased expression.
(XLSX)

S3 Table Pathways populated with ISE6 ortholog proteins following LGTV-infection and UV-LGTV treatment. (XLSX)

S4 Table ISE6 proteins putatively associated with glutaminolysis. (DOCX)

S5 Table *I*. *scapularis* proteins with increased expression following LGTV-infection and UV-LGTV treatment. As mentioned in S1A Fig, four groups of categorized proteins were identified: ISE6 ortholog proteins, ISE6 proteins with no orthology, ISE6 ortholog proteins with no mapped *I*. *scapularis* cellular pathways, and ISE6 ortholog proteins with mapped cellular pathways in other eukaryotes. This table is organized into these four groups including protein cellular function, protein class, and protein pathway. Fold changes of LGTV/mock and UV-LGTV/mock (“nd” denotes not detected) are listed along with search results as to whether the protein has been identified in other flavivirus-host proteomic studies. Proteins listed from Figs 6 and 7 are included in this table with further detail. Fold change of >2 corresponds to an increase expression, 0.5≤fold change≤2 denotes no change in expression, and fold change of <0.5 correlates with decreased expression.
(XLSX)
