# Supplementary figures and images for: Changes in the Proteome of Langat-Infected Ixodes scapularis ISE6 Cells: Metabolic Pathways Associated with Flavivirus Infection
Source: PLoS Negl Trop Dis. 2016 Feb 9;10(2):e0004180. doi: 10.1371/journal.pntd.0004180 (PMC4747643; doi:10.1371/journal.pntd.0004180)

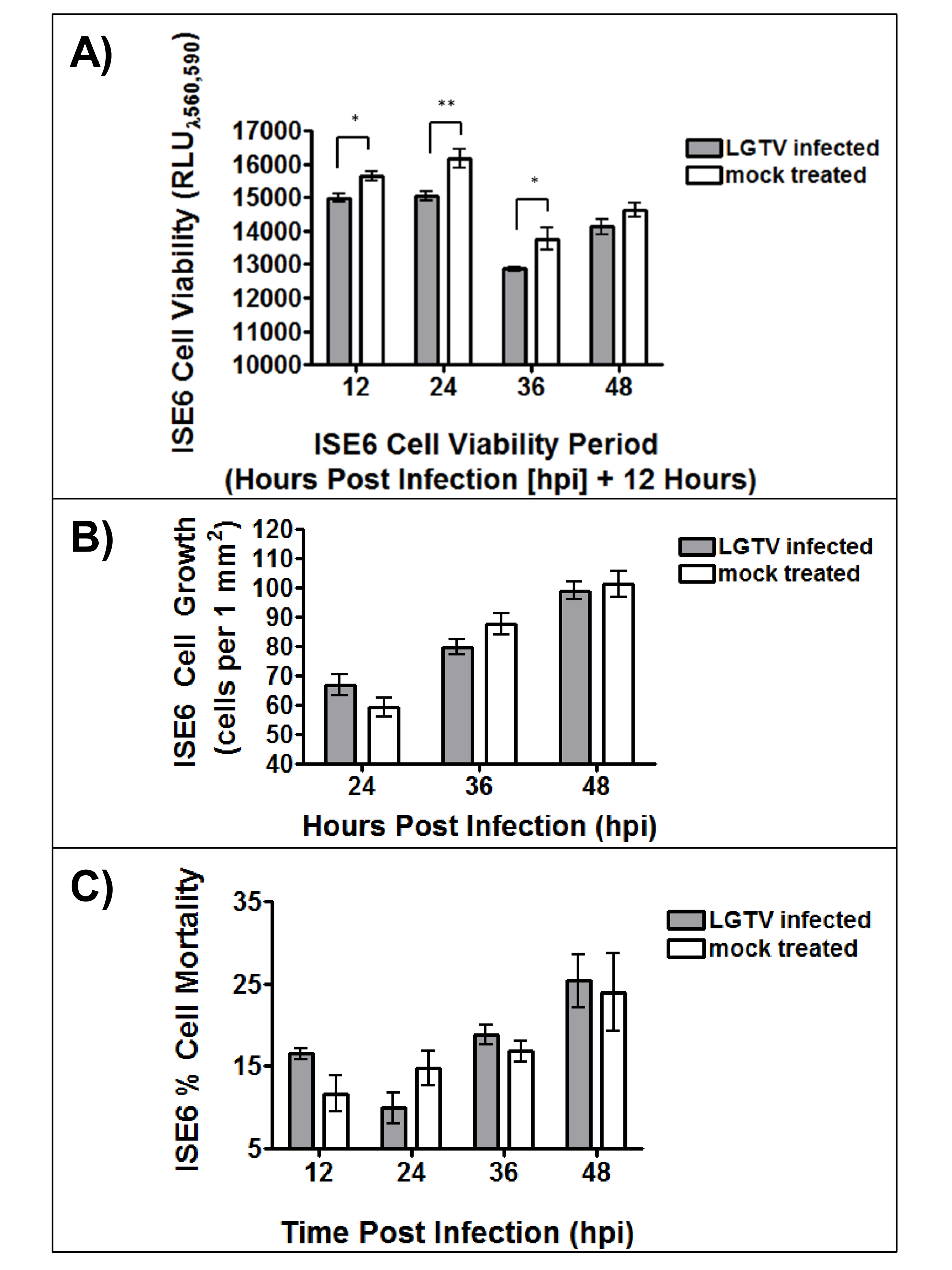

Supplement: S1 Fig — Alteration of ISE6 cell viability (A) at 12–24, 24–36, 36–48 hours post infection are shown in parallel with cell growth/population numbers (B) at 24, 36, 48 hours post infection, and cell mortality percentage (C) at 12, 24, 36, 48 hours post infection with and without LGTV (mock-treated) infection. * denotes p<0.05 and ** denotes p<0.01. Standard error shown in error bars with five technical replicates. Two biological replicate experiments were completed. (TIF) [file pntd.0004180.s001.tif]

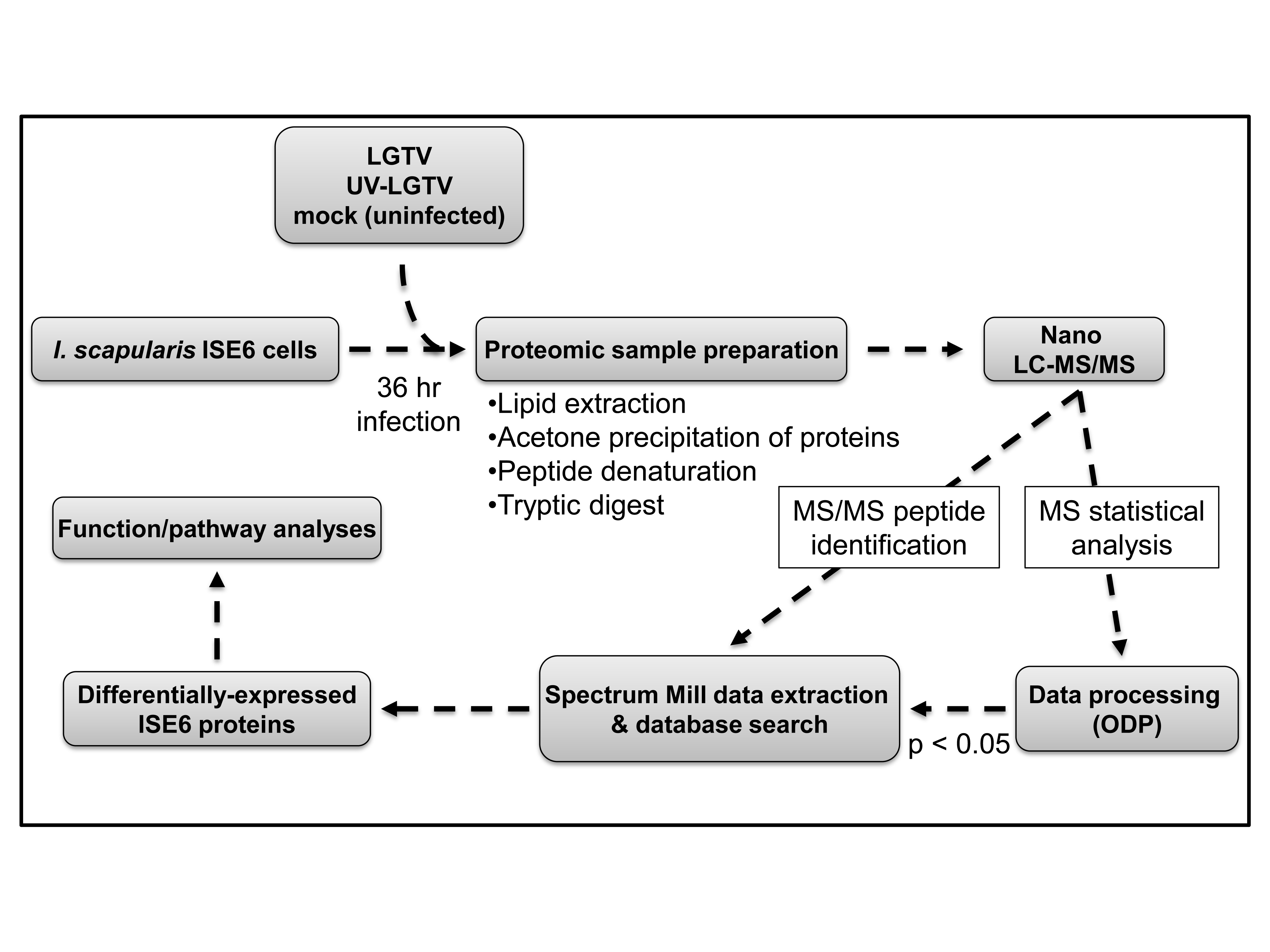

Supplement: S2 Fig — After whole cell sample harvest of treated ISE6 cells, cell pellet samples were subject to lipid removal, protein precipitation, peptide denaturation, and tryptic digest of peptides. Samples were prepared for the separation phase (nano LC) by injection, using electrospray ionization (ESI). Mass analysis of the precursor ion spectra was completed, followed by the second fragment ion MS/MS dimension for downstream peptide identification. Two group and three group statistical analyses with ISE6 cells treated with virus (LGTV), UV-inactivated virus (UV-LGTV), and no virus (mock) were compared utilizing a proteomic/metabolite pipeline, Omics Discovery Pipeline (ODP). After identification of significantly-changing (p < 0.05) MS peaks from LGTV-infected and UV-LGTV-treated ISE6 cells, corresponding peptides were identified to specific I. scapularis proteins (VectorBase I. scapularis WIKEL genome IscaW1.2 predicted protein set database). ISE6 proteins were then subject to protein function and pathway analyses (via KEGG). See materials and methods section for more detail. (TIF) [file pntd.0004180.s002.tif]

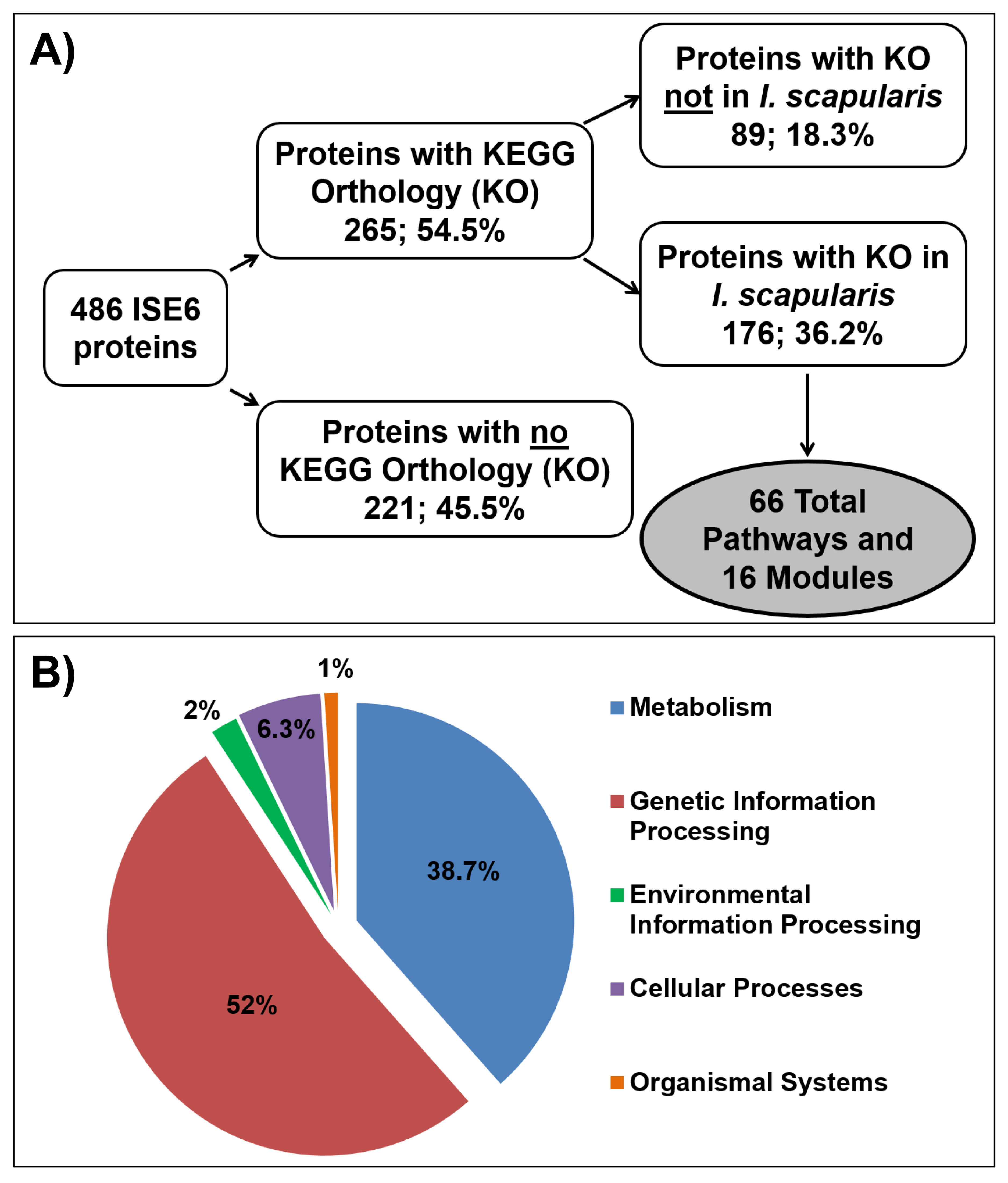

Supplement: S3 Fig — (A) I. scapularis ISE6 proteins with KEGG-mapped orthologs (or KEGG orthology [KO]) help to identify cellular pathways in I. scapularis (genome.jp/kegg/ko). To be identified in a KEGG pathway, KO is required. ISE6 proteins with KO and not identified in I. scapularis (KEGG) pathways are also included. (B) Percent cellular function distribution of proteins found in the 66 identified I. scapularis (KEGG) pathways with 16 modules. (TIF) [file pntd.0004180.s003.tif]

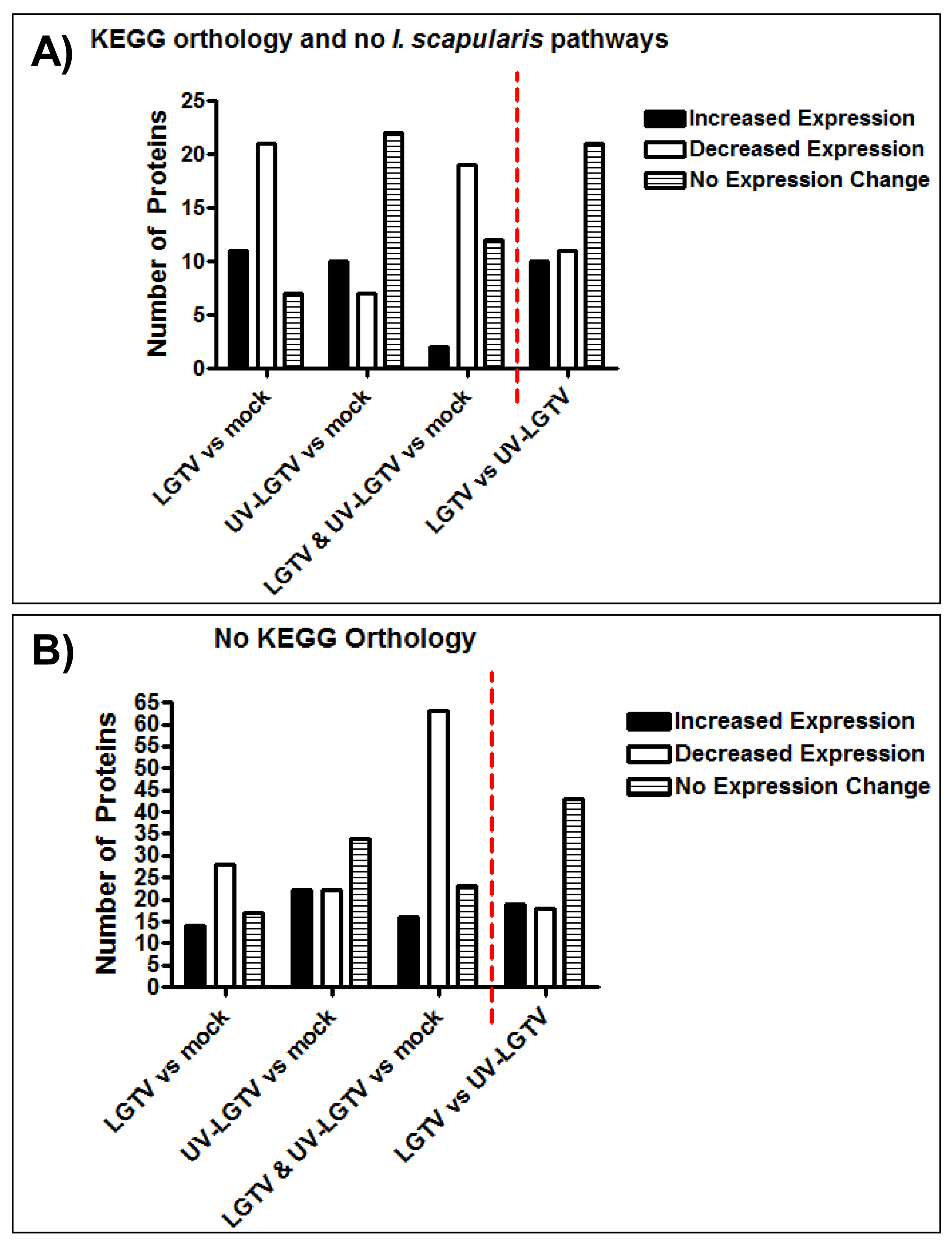

Supplement: S4 Fig — Expression of ISE6 proteins with (A) or without (B) orthology and no identified pathways. Refer to S2 Table for more specifics on the proteins. Red dotted line denotes differentially-expressed proteins in LGTV-infected ISE6 cells compared to UV-LGTV-treated ISE6 cells (no comparison to mock-treated ISE6 cells). (TIF) [file pntd.0004180.s004.tif]

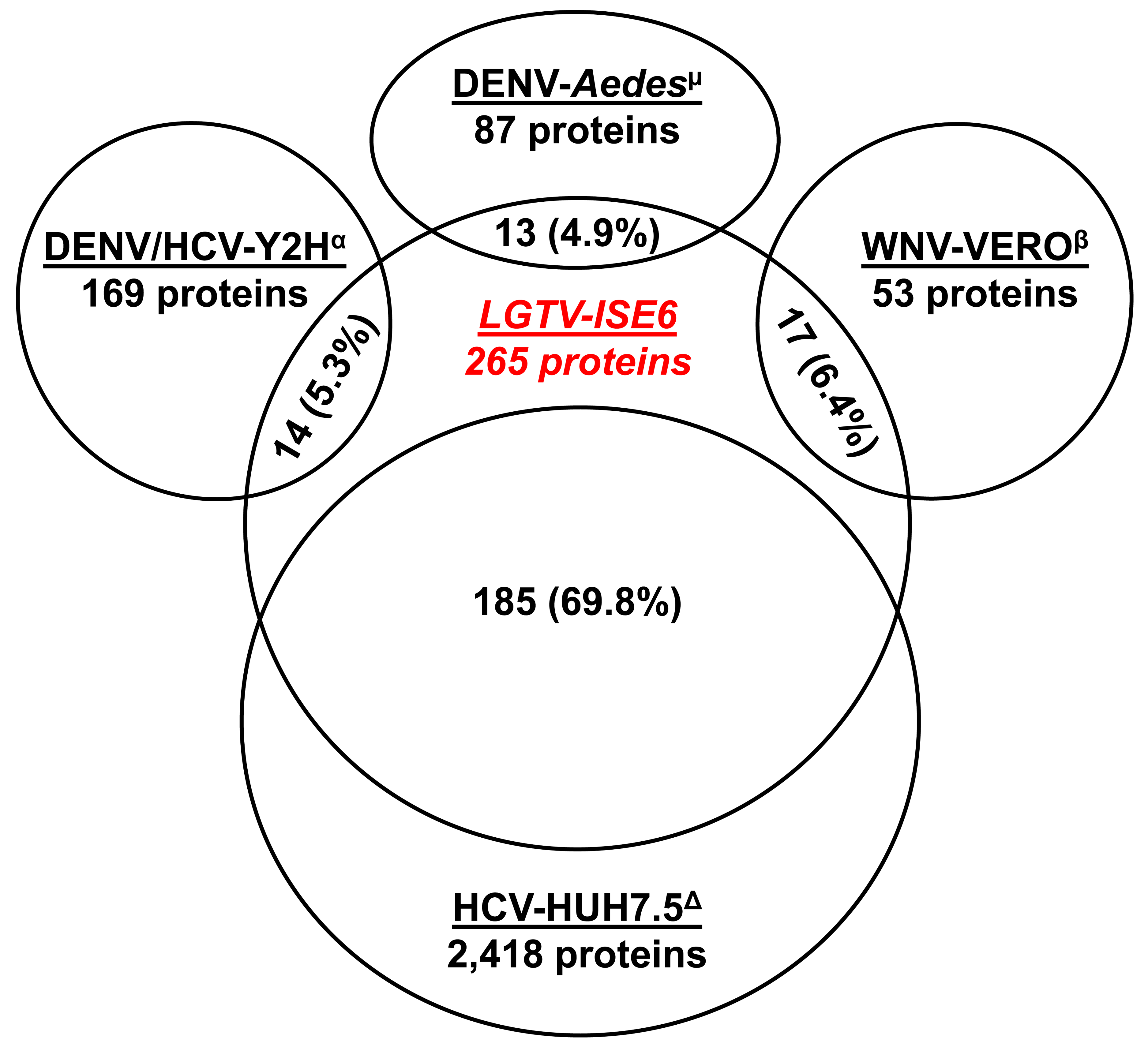

Supplement: S5 Fig — Corresponding percentages correspond to the number of tick ISE6 orthologs identified with orthologs identified in: α S5 Fig, S7 Fig, and S11 Fig of Khadka et al. [56]; μS2 Table of Tchankouo-Nguetcheu et al. [28]; βTables 1 and 2 of Pastorino et al.[55]; ΔS1 Table of Diamond et al.[19]. (TIF) [file pntd.0004180.s005.tif]
