# Supplementary material for: Changes in the Proteome of Langat-Infected Ixodes scapularis ISE6 Cells: Metabolic Pathways Associated with Flavivirus Infection
Source: PLoS Negl Trop Dis. 2016 Feb 9;10(2):e0004180. doi: 10.1371/journal.pntd.0004180 (PMC4747643; doi:10.1371/journal.pntd.0004180)
Supplement: S1 Table — (DOCX) [file pntd.0004180.s006.docx]

**S1 Table**. **Summary of analyses used to identify proteins** **from LGTV-infected and UV-LGTV-treated ISE6 cell samples**

| **Processing Step** | | **No MS peaks/Peptides/Proteins per Treatment** | | | |
| --- | --- | --- | --- | --- | --- |
|  | **Description** | **LGTV vs. mock** | **UV-LGTV vs. mock** | **LGTV vs. UV-LGTV** | **LGTV vs. mock vs. UV-LGTV** |
| **MS Data (Number of Peaks)** | | | | | |
| Total number of LC-MS peaks observed | LC-MS data were aligned and normalized using ODP^a^ | 39,241 | 37,218 | 31,364 | 46,590 |
| Number of LC-MS peaks with corresponding peptides | All normalized peaks that matched to peptides | 27,868 | 30,789 | 22,054 | 33,117 |
| Number of significant LC-MS peaks between treatment groups | The ODP was used to calculate a P value based on the intensities of each LC-MS peak between groups^b^ | 14,658  T-test (P< 0.05) | 18,759  T-test (P< 0.05) | 5,247  T-test (P< 0.05) | 18,402  ANOVA (P< 0.05) |
| Data filtering based on Spectrum Mill score | Peptide score^3^ (> 5) and removal of unidentified LC-MS peaks | 8,154 | 10,267 | 2,898 | 9,507 |
|  | % scored peak intensity^c^ (%SPI≥ 70%) | 4,781 | 5,878 | 1,727 | 5,516 |
| Number of peaks following removal of peptide false positives | Δ fwd-rev score^d^ > 1 with parent charges +1 and +2  Δ fwd-rev score > 2 with parent charges +3 and +4 | 4,310 | 5,237 | 1,540 | 4,914 |
| Final List of MS peaks | Removal of replicate MS peaks identified in the multiple samples | 1,096 | 1,355 | 356 | 1,267 |
| **Number of Peptides** | | | | | |
| Peptides | Number of unique peptides | 772 | 893 | 315 | 821 |
| **Number of Proteins** | | | | | |
| Proteins | Proteins identified  to VectorBase accession ISCW-IDs^e^ | 374 | 409 | 201 | 395 |

**^a^**ODP denotes Omics Discovery Pipeline

^b^After normalization of MS peaks in each treatment group (LGTV, UV-LGTV, and mock), t-test and ANOVA analyses were performed in the Significance Test step of the ODP.

^c^Spectrum Mill peptide score and % scored peak intensity are two criteria utilized to identify peptides from *in silico* database searches. See material and methods section for more details.

^d^Spectrum Mill reverse database search was completed to account for possible false positives when identifying peptides from *in silico* database searches. See material and methods section for more details.

^e^≥1 peptide match to a VectorBase gene model predicted peptide sequence was considered a protein identification. See material and methods section for more details.
