# Supplementary material for: Changes in the Proteome of Langat-Infected Ixodes scapularis ISE6 Cells: Metabolic Pathways Associated with Flavivirus Infection
Source: PLoS Negl Trop Dis. 2016 Feb 9;10(2):e0004180. doi: 10.1371/journal.pntd.0004180 (PMC4747643; doi:10.1371/journal.pntd.0004180)
Supplement: S4 Table — (DOCX) [file pntd.0004180.s009.docx]

**S4 Table. ISE6 proteins putatively associated with glutaminolysis.**

| **Protein**  **(VB accession name)** | **KEGG ortholog** | **Putative functions**  **(GO predictions)** | **Orthology to glutaminolytic enzyme** | **Change in expression** |
| --- | --- | --- | --- | --- |
| (ISCW011739) prostate-specific transglutaminase, putative | NP* | - peptide cross-linking - protein-glutamine gamma-glutamyltransferase activity - transferase activity, transferring acyl groups - transferase activity | Glutaminase  (Glase) | Increased expression with LGTV |
| (ISCW000393) glutamate dehydrogenase, putative | K00261  glutamate  dehydrogenase (NAD(P)+) | - cellular amino acid metabolic process - oxidation-reduction process - oxidoreductase activity, acting on the CH-NH2 group of donors, NAD or NADP as acceptor - oxidoreductase activity | Glutamate dehydrogenase  (GDH) | Increased expression with both LGTV and UV-LGTV |
| (ISCW015982) aldehyde dehydrogenase, putative | K00294  1-pyrroline-5-carboxylate dehydrogenase | - proline biosynthetic process - oxidation-reduction process - metabolic process - oxidoreductase activity, acting on the aldehyde or oxo group of donors, NAD or NADP as acceptor - 1-pyrroline-5-carboxylate dehydrogenase activity - oxidoreductase activity | GDH | Increased expression with both LGTV and UV-LGTV |
| (ISCW001521) membrane protein,  putative | NP | - oxidation-reduction process - saccharopine dehydrogenase (NAD+, L-glutamate-forming) activity - oxidoreductase activity | GDH | Increased expression with both LGTV and UV-LGTV |
| (ISCW020361) glutamine synthetase 1, putative | NP | - nitrogen compound metabolic process - glutamine biosynthetic process - ligase activity - glutamate-ammonia ligase activity |  | Decreased expression with both LGTV and UV-LGTV |

*****NP denotes does not have a KEGG Ortholog.
